# FAM188B Downregulation Sensitizes Lung Cancer Cells to Anoikis via EGFR Downregulation and Inhibits Tumor Metastasis In Vivo

**DOI:** 10.3390/cancers13020247

**Published:** 2021-01-11

**Authors:** Eun-Ju Jang, Jee Young Sung, Ha-Eun Yoo, Hyonchol Jang, Jaegal Shim, Eok-Soo Oh, Sung-Ho Goh, Yong-Nyun Kim

**Affiliations:** 1 Division of Translational Science, National Cancer Center, 323 Ilsan-ro, Ilsandong-gu, Goyang-si 10408, Gyeonggi-do, Korea; eunju2190@ncc.re.kr (E.-J.J.); Sungjy@ncc.re.kr (J.Y.S.); he3756@ncc.re.kr (H.-E.Y.); jaegal@ncc.re.kr (J.S.); 2 Department of Life Sciences, Ewha Womans University, 52, Ewhayeodae-gil, Seodaemun-gu, Seoul 120-750, Korea; ohes@ewha.ac.kr; 3 Division of Cancer Biology, National Cancer Center, 323 Ilsan-ro, Ilsandong-gu, Goyang-si 10408, Gyeonggi-do, Korea; hjang@ncc.re.kr; 4 Division of Precision Medicine, National Cancer Center, 323 Ilsan-ro, Ilsandong-gu, Goyang-si 10408, Gyeonggi-do, Korea

**Keywords:** FAM188B, EGFR, anoikis

## Abstract

**Simple Summary:**

Cancer cells should acquire anoikis resistance for successful metastasis. Family with sequence similarity 188 member B (FAM188B) has been identified as a new deubiquitinase (DUB) member. Here, we demonstrate that FAM188B knockdown makes lung cancer cells sensitive to anoikis and inhibits lung metastasis. FAM188B knockdown reduced the levels of tumor proteins such as EGFR and FOXM1, suggesting that FAM188B may be a potential target controlling tumor malignancies.

**Abstract:**

Anoikis is a type of apoptosis induced by cell detachment from the extracellular matrix (ECM), which removes mislocalized cells. Acquisition of anoikis resistance is critical for cancer cells to survive during circulation and, thus, metastasize at a secondary site. Although the sensitization of cancer cells to anoikis is a potential strategy to prevent metastasis, the mechanism underlying anoikis resistance is not well defined. Although family with sequence similarity 188 member B (FAM188B) is predicted as a new deubiquitinase (DUB) member, its biological function has not been fully studied. In this study, we demonstrated that FAM188B knockdown sensitized anoikis of lung cancer cell lines expressing WT-EGFR (A549 and H1299) or TKI-resistant EGFR mutant T790M/L858R (H1975). FAM188B knockdown using si-FAM188B inhibited the growth of all three human lung cancer cell lines cultured in both attachment and suspension conditions. FAM188B knockdown resulted in EGFR downregulation and thus decreased its activity. FAM188B knockdown decreased the activities of several oncogenic proteins downstream of EGFR that are involved in anoikis resistance, including pAkt, pSrc, and pSTAT3, with little changes to their protein levels. Intriguingly, si-FAM188B treatment increased EGFR mRNA levels but decreased its protein levels, which was reversed by treatment with the proteasomal inhibitor MG132, indicating that FAM188B regulates EGFR levels via the proteasomal pathway. In addition, cells transfected with si-FAM188B had decreased expression of FOXM1, an oncogenic transcription factor involved in cell growth and survival. Moreover, FAM188B downregulation reduced metastatic characteristics, such as cell adhesion, invasion, and migration, as well as growth in 3D culture conditions. Finally, tail vein injection of si-FAM188B-treated A549 cells resulted in a decrease in lung metastasis and an increase in mice survival in vivo. Taken together, these findings indicate that FAM188B plays an important role in anoikis resistance and metastatic characteristics by maintaining the levels of various oncogenic proteins and/or their activity, leading to tumor malignancy. Our study suggests FAM188B as a potential target for controlling tumor malignancy.

## 1. Introduction

Tumor metastasis is responsible for more than 90% of cancer-related deaths [1]. Tumor metastasis involves various series of sequential steps, such as dissociation of cancer cells from primary tumor; invasion of cancer cells into the extracellular matrix (ECM); intravasation of cancer cells into the circulation system; cell survival during circulation, extravasation, and adaptation of cancer cells to a new environment to settle down in the secondary site; and proliferation in new microenvironments to form secondary tumors. Once cancer cells enter the bloodstream, these circulating tumor cells can overcome various adversities, such as shear stress and anoikis, resulting in their survival [2,3].

Anoikis is a type of apoptosis activated upon cell detachment from the extracellular matrix (ECM), and normal epithelial cells usually undergo anoikis when they become detached from the ECM [4]. However, cancer cells become resistant to anoikis and, thus, survive under anchorage-independent conditions such as circulation via the bloodstream without ECM attachment. Because circulating tumor cells (CTCs) are the main causative cells for distant metastasis and only viable CTCs can be metastasized, it is very important to elucidate mechanisms underlying anoikis resistance [5]. Studies have demonstrated that activation of survival signaling is important for anoikis resistance. For example, activation of EGFR inhibits the accumulation of Bim, which is a pro-apoptotic molecule, and thus prevents mitochondrial-pathway-mediated anoikis when cells are detached from the ECM [6,7]. We have reported that cell detachment upregulates NADPH oxidase 4 (NOX4), which maintains EGFR levels and activities that are important for anoikis resistance in human lung cancer cells. STAT3 is an oncogenic transcription factor that enhances genes involved in the cell cycle, such as cyclin D1, and anti-apoptotic proteins such as Survivin, Bcl-2, and Bcl-xL [8,9]. STAT3 activation has recently been shown to mediate anoikis resistance and enhance invasive properties to nasopharyngeal cancer cells [10]. Although several pathways for anoikis resistance have been reported, much still remains to be explored to understand the mechanisms for the acquisition of anoikis resistance.

Recently, family with sequence similarity 188 member B (FAM188B), also known as MINDY-4, has been reported as an evolutionarily distant member of the FAM63A protein (MINDY-1) [11], which is a putative deubiquitinase that cleaves the Lys-48-linked polyubiquitin chain. Although this novel protein FAM188B is annotated as a “probable ubiquitin carboxyl-terminal hydrolase” in the database [12], its function has not yet been demonstrated. We previously reported that FAM188B knockdown results in cell death via accumulation and activation of p53 in colorectal cancer cell lines [13], indicating that FAM188B expression is important for cell survival. In addition, we found that FAM188B expression is enhanced and poorly correlated with poor survival in lung cancer patients [14], indicating that FAM188B might have a role in tumorigenesis and malignancy. In this study, we evaluated, for the first time, the importance of FAM188B in the regulation of anoikis resistance. Here, we demonstrate that FAM188B knockdown sensitizes human lung cancer cell lines to anoikis upon cell detachment and inhibits lung metastasis in vivo. FAM188B knockdown induced EGFR downregulation and inactivation of survival-related signaling molecules, indicating that FAM188B could be a potential target to control tumor metastasis.

## 2. Result

**Figure 1:**
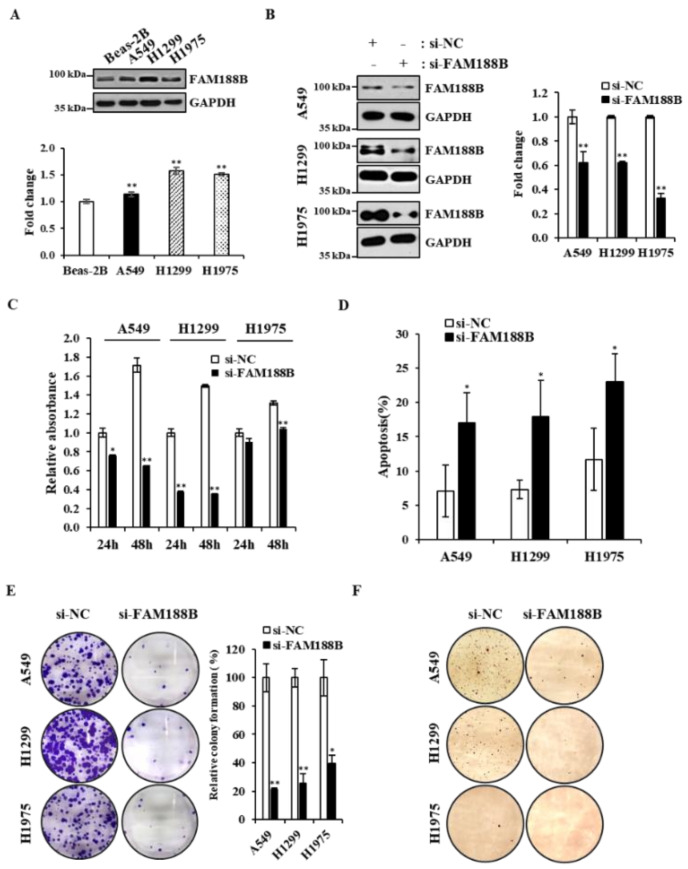
**Effects of FAM188B knockdown on cell viability in lung cancer cells** (**A**) Beas-2B, A549, H1299, and H1975 cells were processed for immunoblot analysis using indicated antibodies (upper panel). The levels family with sequence similarity 188 member B (FAM188B) were quantified by densitometry and normalized to Beas-2B (lower panel). GAPDH was used as a loading control. (* *p* < 0.05, ** *p* < 0.01). (**B**–**D**) A549, H1299, and H1975 cells were transiently transfected with either nonspecific control si-RNA (si-NC) or si-RNA targeting FAM188B (si-FAM188B) for 48 h, followed by immunoblot analysis using indicated antibodies (left panel) and the levels FAM188B were quantified by densitometry and normalized to GAPDH (right panel) (**B**), cell growth analysis by 3-(4,5-dimethylthiazol-2-yl)-5-(3-carboxyme-thoxyphenyl)-2-(4-sulfophenyl)-2H-tetrazolium (MTS) (**C**), and apoptosis assay of annexin V/propidium iodide (PI)-stained cells (**D**). (**E**) Cells were transiently transfected with either si-NC or si-FAM188B for 24 h, followed by colony forming assays as described in the “Materials and Methods”. Representative images are shown (left panel), and the relative colony formation was quantified using Image J (right panel). (**F**) Cells transfected as in (**E**) were processed for soft agar assay as described in the “Materials and Methods,” and representative images are shown. Error bars represent standard deviations of the mean of three measurements (* *p* < 0.05, ** *p* < 0.01, versus si-NC). These experiments were performed three times independently with similar results.

**FAM188B knockdown decreased cell viability in lung cancer cells**. First, we examined basal levels of FAM188B protein in a normal human lung epithelial cell line, Beas-2B, and three human lung cancer cell lines, A549, H1299, and H1975, by immunoblot analysis. A549 and H1299 cells express WT-EGFR, and H1975 cells express T790M/L858R mutant EGFR that is EGFR-TKI-resistant [15]. As shown in Figure 1A, protein levels of FAM188B were higher in the lung cancer cell lines than in normal Beas-2B cells. Next, we tested whether FAM188B expression is important for cell growth by knockdown of FAM188B using si-RNA against FAM188B. FAM188B was downregulated by si-RNA targeting FAM188B (Figure 1B), and cell growth was significantly inhibited by FAM188B knockdown as assessed by 3-(4,5-dimethylthiazol-2-yl)-5-(3-carboxyme-thoxyphenyl)-2-(4-sulfophenyl)-2H-tetrazolium (MTS) assay in A549, H1299, and H1975 cells (Figure 1C). To test whether this FAM188B knockdown-induced growth inhibition is due to cell death, we performed apoptosis analysis of annexin V/propodium iodine (PI)-stained cells by flow cytometry. There were increases in annexin V/PI-stained cells by si-FAM188B-treatment, indicating that FAM188B knockdown induces apoptosis (Figure 1D and Figure S1A). FAM188B knockdown induced increased caspase 3 activation, as assessed by its cleavage, and thus increased PARP cleavage (Figure S1B), indicating activation of apoptosis signaling by FAM188B knockdown. In addition, colony formation ability was reduced by si-FAM188B transfection in all three cell lines (Figure 1E). Furthermore, although there were some variations in the ability to form colonies among the cell lines, FAM188B knockdown decreased colony formation in soft agar conditions, which is a measure of anchorage-independent growth (Figure 1F). These data indicate that FAM188B expression is important for cell growth in lung cancer cell lines.

**Figure 2:**
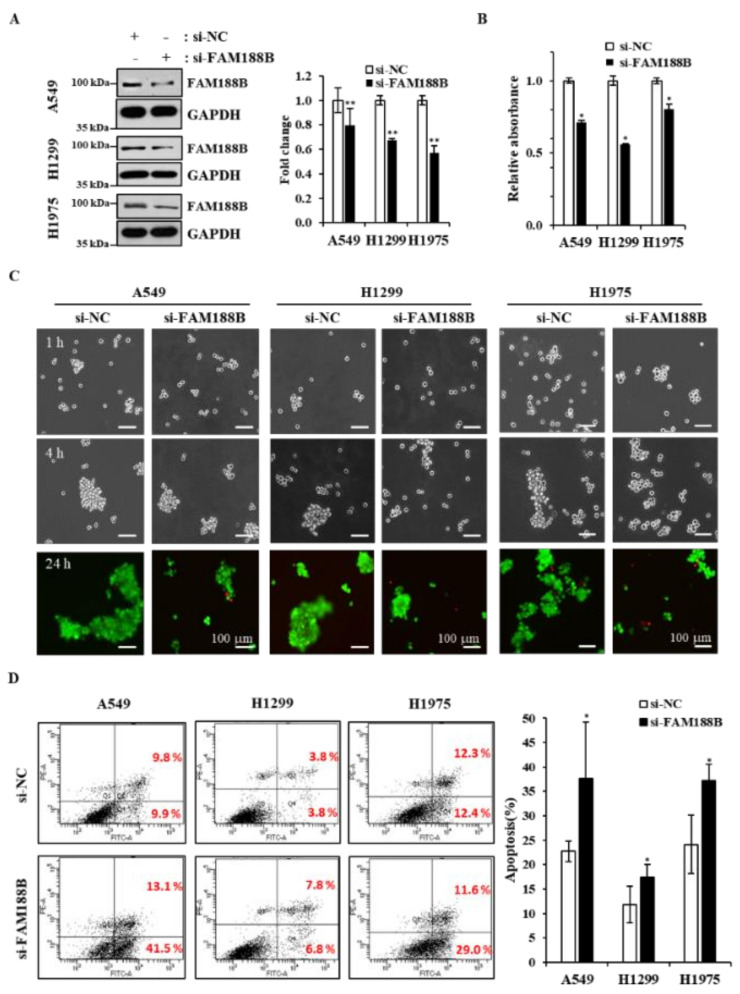
**Effects of FAM188B knockdown on cell aggregation and apoptosis upon cell detachment.** (**A**–**D**) Cells were transiently transfected with either si-NC or si-FAM188B for 48 h, and then cells were grown in the HEMA-coated plate for suspension culture for 24 h. Cells were then processed for immunoblot analysis using indicated antibodies (**A**), cell growth analysis by MTS (**B**), calcein- acetoxymethyl (AM)/PI staining for live/dead cell assay at indicated times (**C**), or annexin V/PI staining for apoptosis assay (**D**) as described in the “Materials and Methods”. The levels FAM188B were quantified by densitometry and normalized to GAPDH (**A**, right panel). Data are presented as mean values from three measurements, and error bars represent standard deviations. (* *p* < 0.05, ** *p* < 0.01 versus si-NC). Scale bars = 100 μm. These experiments were performed three times independently with similar results.

**FAM188B knockdown sensitizes anoikis of lung cancer cells**. Because FAM188B appeared to be important for anchorage-independent growth, we further investigated whether FAM188B expression affects cell survival in suspension culture where cells grow without adhesion to the plate. Cells were transfected with si-FAM188B and cultured in poly-HEMA-coated plates to prevent cells from adhesion (Figure 2A). Cell growth decreased due to FAM188B knockdown in suspension culture in all three cell lines as determined by MTS (Figure 2B). Previously, we and others have demonstrated that the formation of multicellular aggregates is important for anoikis resistance in suspension culture [16,17]. When cells were cultured in suspension, cells formed aggregates in a time-dependent manner. However, FAM188B knockdown inhibited cell aggregate formation in all three cell lines with an increase in dead cells stained by PI and shown in red (Figure 2C). Although we used si-FAM188B RNA that has been described in our previous report [14], we also used other si-FAM188B RNAs to avoid off-target effects. As shown in Figure S2, all three si-FAM188B RNAs reduced cell aggregate formation in the suspension culture. We also observed that FAM188B knockdown induced apoptosis in the suspension culture as determined by flow cytometry analysis of annexin V/PI stained cells (Figure 2D). All these data indicate that FAM188B knockdown sensitizes cancer cells to anoikis when cultured in suspension.

**Figure 3:**
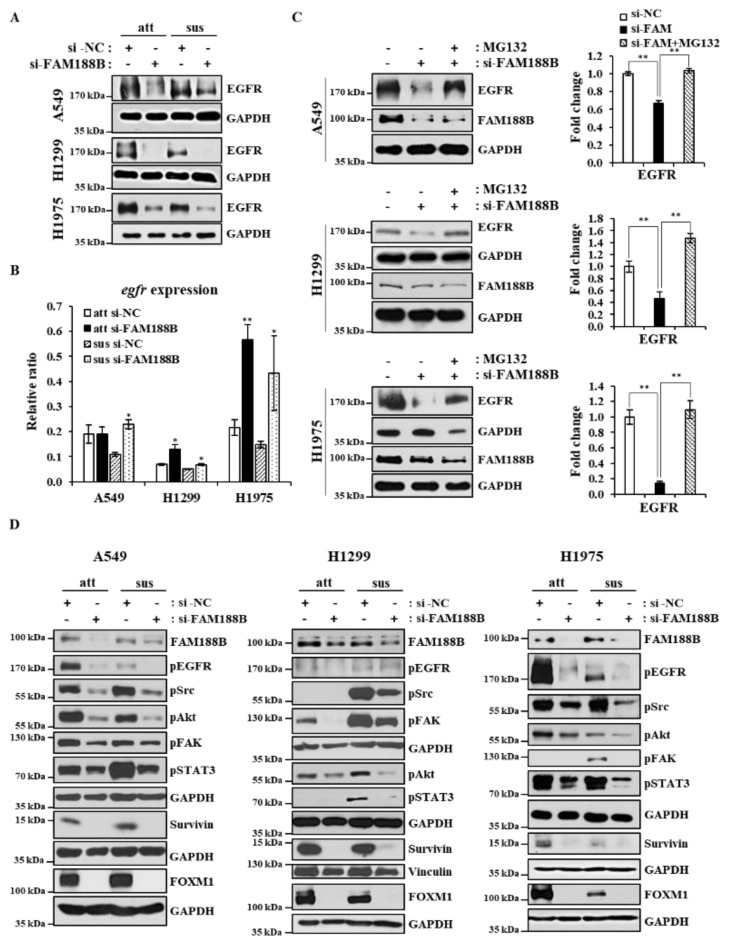
**Effects of FAM188B knockdown on cell EGFR levels and survival signaling pathways.** (**A**,**B**,**D**) Cells were transiently transfected with either si-NC or si-FAM188B for 48 h, followed by either attached or suspended culture for 24 h. Cells were then processed for immunoblot analysis using indicated antibodies. The levels of proteins were quantified by densitometry and normalized to GAPDH or Vinculin (Figure S3B–E) (**A**,**D**). qRT-PCR assay for relative EGFR mRNA expression levels (**B**). GAPDH was used as a loading control. (**C**) Cells were transiently transfected with either si-NC or si-FAM188B for 48 h and then treated with 20 μM MG132 for 2 h before harvest. Cell lysates were processed for immunoblot analysis using indicated antibodies (left panel), and the levels of EGFR were quantified by densitometry and normalized to GAPDH (right panel). Error bars represent standard deviations of the mean of three measurements (* *p* < 0.05, ** *p* < 0.01). These experiments were performed three times independently with similar results.

**FAM188B regulates EGFR through the stabilization of EGFR expression.** We and others have reported that EGFR activation is important for anoikis resistance. In addition, EGFR plays a critical role in the progression of tumor malignancy, including survival and invasion, and thus facilitating tumor metastasis [18]. Interestingly, FAM188B knockdown downregulated EGFR protein levels in both attached and suspended cells regardless of EGFR mutation (Figure 3A). More interestingly, FAM188B re-expression could recover EGFR receptor levels that had been decreased by FAM188B knockdown (Figure S3A). To test whether EGFR is downregulated at the transcriptional levels, we performed qRT-PCR of EGFR after FAM188B knockdown. Contrary to expectation, FAM188B knockdown upregulated EGFR mRNA levels (Figure 3B). Next, we tested whether FAM188B regulates EGFR at the level of protein stability by using a proteasome inhibitor, MG132. MG132 treatment could restore EGFR levels in the si-FAM188B-transfected cells (Figure 3C), indicating that FAM188B regulates EGFR levels via stabilization. EGFR activates various signaling pathways, such as Src, Akt, and STAT3, which are known to be involved in anoikis resistance [19,20,21]. Intriguingly, FAM188B knockdown inactivated Src, Akt, and STAT3, not only in suspension but also in attached cells (Figure 3D). FAK activation is also important for anoikis resistance [22], and FAK phosphorylation was reduced by FAM188B knockdown (Figure 3D). More interestingly, FAM188B knockdown decreased FOXM1, an oncogenic transcription factor [23], in both attached and suspended conditions (Figure 3D). Survivin is a pro-survival protein that is known to be transcriptionally regulated by STAT3 and FOXM1 [24,25]. Survivin was also reduced in the FAM188B-knockdown cells (Figure 3D). All these data suggest that FAM188B knockdown downregulates EGFR levels through the proteasome degradation pathway and inactivates multiple pathways involved in cell proliferation and survival.

**Figure 4:**
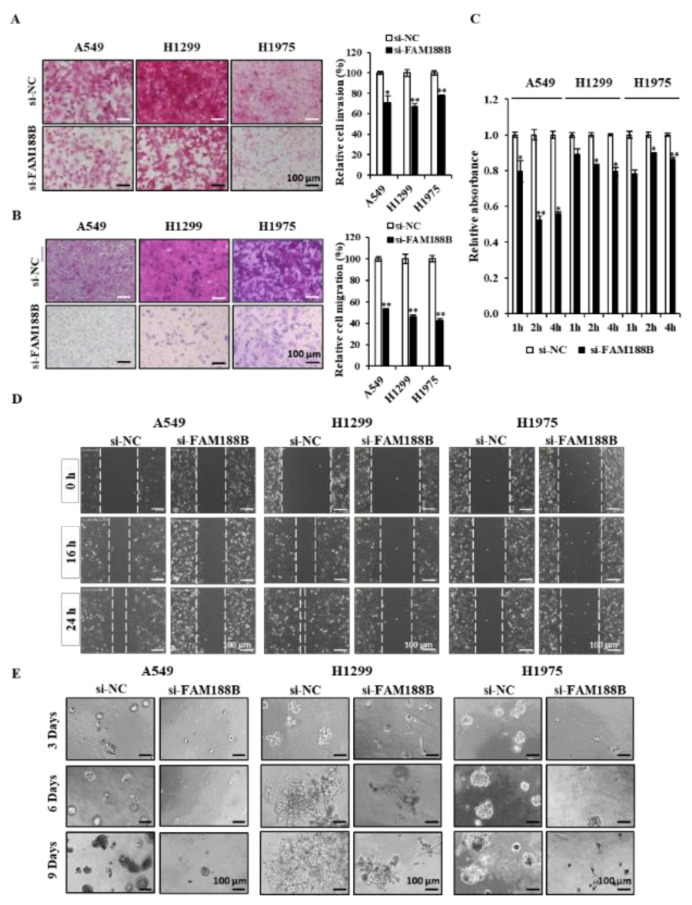
**Effects of FAM188B knockdown on several metastatic characteristics in lung cancer cell lines.** (**A**–**E**) Cells were transiently transfected with either si-NC or si-FAM188B for 24 h, followed by invasion assay (**A**), migration assay (**B**), adhesion assay (**C**), wound healing assay (**D**), and 3D culture (**E**) as described in the “Materials and Methods” and representative images are shown. For relative invasion and migration analysis, images were scanned to quantify three different areas (right panels in **A**,**B**). For adhesion assay, 5 × 10^3^ cells were plated in 96-well plate, and at indicated times, floating cells were removed by washing, and attached cells were measured by MTS assay (**C**). For wound healing assay, confluent cells were grown in 12-well plate, and cell monolayers were scratched with a pipet tip, and then cells were incubated for 24 h (**D**). For 3D culture, 5 × 10^3^ cells in 400 μL of 5% Matrigel were plated in the Matrigel-coated 8-well chamber as described in the “Materials and Methods”. Colony images were captured on indicated days (**E**). Scale bars = 100 μm. Error bars represent standard deviations of the mean of three measurements (* *p* < 0.05, ** *p* < 0.01, versus si-NC). These experiments were performed three times independently with similar results.

**FAM188B knockdown inhibits various metastatic characteristics and tumor metastasis.** Tumor metastasis involves various processes, including cell migration, invasion, cell deadhesion from the ECM, and re-adhesion to the ECM to settle and proliferate at the secondary site [26]. We tested whether knockdown of FAM188B inhibits these metastatic features in three lung cancer cell lines. FAM188B knockdown significantly decreased both invasion (Figure 4A) and migration (Figure 4B) in three cell lines. In addition, cell adhesion was also retarded in cells treated with si-FAM188B (Figure 4C). FAM188B knockdown decreased wound closure in all three cell lines (Figure 4D). We further examined whether FAM188B could affect cancer cell growth in the 3D culture condition, which mimics in vivo environments [27]. Knockdown of FAM188B not only decreased the sizes of colonies but also altered the shape of growth when they were grown in 3D culture (Figure 4E). These data indicate that FAM188B expression is important for the regulation of metastatic features.

**Figure 5:**
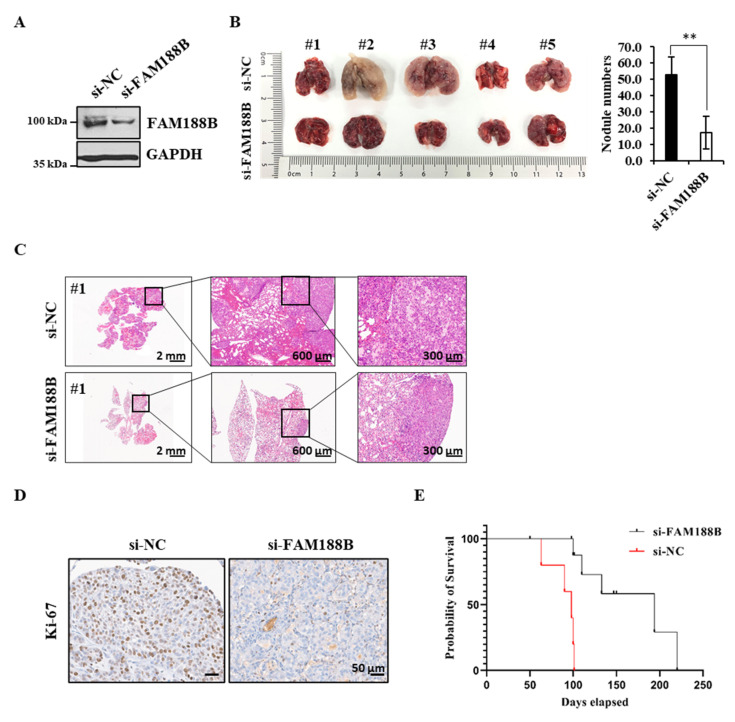
**In vivo effects of FAM188B knockdown on lung metastasis and survival in xenograft mouse model.** (**A**–**E**) A549 cells were transiently transfected with either si-NC or si-FAM188B for 48 h, and then cells were injected to BALB/c nude mice (*n* = 10) via the tail vein. FAM188B knockdown in A549 cells was verified by immunoblotting and the levels of FAM188B were quantified by densitometry and normalized to GAPDH (Figure S4C) (**A**). The mice were sacrificed after eight weeks, lung images were shown (left panel), and the number of tumor nodules in the lungs was counted (right panel) (**B**). Representative pictures of hematoxylin and eosin staining of lung tissues were shown. Scale bars = 2 mm, 600 μm, and 300 μm (**C**). Lung tissues were processed for immunohistochemistry with anti-Ki-67 antibodies. Scale bars = 50 μm (**D**) Kaplan–Meier survival curves of mice bearing either si-NC-A549 cells or si-FAM188B-A549 cells (**E**). (** *p* < 0.01, versus si-NC) Similar results were observed in two independent experiments.

To further investigate the role of FAM188B in metastasis in vivo, A549 cells with or without si-FAM188B were injected into the tail vein of BALB/c nude mice, and the occurrence of pulmonary metastasis was examined (Figure 5A). Interestingly, upon treatment with si-FAM188B, the degree of lung metastasis determined by the number of tumor nodules was reduced when compared with nonspecific control si-RNA (si-NC) treatment (Figure 5B). Consistent with these observations, tumor metastasis decreased as assessed by lung histology via hematoxylin and eosin staining (Figure 5C and Figure S4A). When tumor tissues were analyzed by immunohistochemistry using the cell proliferation marker Ki-67, Ki-67 levels decreased in the si-FAM188B group compared with the si-NC group (Figure 5D). STAT3 activation was dramatically reduced by FAM188B knockdown (Figure 3D), and consistently phospho-STAT3 immunostaining decreased in the FAM188B-knockdown lung tumor tissue (Figure S4B). Finally, the mean survival times for mice with si-NC and si-FAM188B were 100 and 220 days, respectively. The mice with si-FAM188B exhibited a prolonged survival time compared with si-NC mice (*p* < 0.001; Figure 5E). Taken together, our data indicate that FAM188B expression is important for cell survival, especially anoikis resistance, which is critical for cancer metastasis.

## 3. Discussion

Metastasis plays a critical role in cancer-related mortality [28]. Anoikis is a type of cell death caused by cell detachment from the ECM, which prevents the dissemination of mis-localized cells [29]. For successful metastasis in distant and vital organs, cancer cells must acquire anoikis resistance, which is an essential property for cancer cells to survive in the blood circulation or lymphatic system [30]. However, the molecular mechanisms involved in cancer cell anoikis resistance are not fully defined. In this study, we demonstrate, for the first time, that the expression of FAM188B is important for the regulation of anoikis and the development of metastatic properties in human lung cancer cell lines.

Anoikis is a form of cell death initiated by cell detachment from the ECM, thus preventing the attachment of the detached cells to other organs [29]. Anoikis is a major hindrance that cancer cells must overcome for successful metastasis [2]. Most CTCs are known to be anoikis-sensitive, but some of them become anoikis-resistant and metastasize at secondary sites [5,31]. Therefore, an understanding of the molecular mechanisms behind anoikis resistance would provide new therapeutic targets to prevent tumor metastasis. In this study, we show that FAM188B expression is important for anoikis resistance. Consistent with a previous report using colon cancer cells [13], FAM188B knockdown inhibited the growth of lung cancer cell lines regardless of whether they were expressing wild type or mutant EGFR (Figure 1C,D). In addition, FAM188B knockdown decreased the growth of lung cancer cell lines in soft agar (Figure 1F), and FAM188B knockdown sensitized cells to anoikis when cells were in suspension (Figure 2C,D). These data indicate that FAM188B expression is important for anchorage-independent growth and anoikis resistance, which are critical properties of tumor malignancy [32].

EGFR is a well-known proto-oncogene, and its hyperactivation via mutation and overexpression has been linked to tumor development and malignancy in various solid tumors, including lung cancer, breast cancer, and colon cancer [33]. EGFR enhances cell proliferation, migration, invasion, and survival and thus contributes to tumor progression [34]. It has been demonstrated that EGFR overexpression prevents cells from undergoing anoikis via degradation of a pro-apoptotic protein, Bim [35]. Hepatocellular-carcinoma-related protein-1 (HCRP-1) is known to be downregulated in various malignant tumors, and its downregulation induces anoikis resistance via Bim downregulation in the EGFR–Akt pathway [36]. Previously, we also reported that maintenance of EGFR levels and activity is critical for escaping anoikis in A549 lung cancer cells [37]. In this regard, it is very intriguing that FAM188B knockdown decreased levels of total EGFR and p-EGFR in both attached and detached cells (Figure 3A,D). Interestingly, FAM188B knockdown did not decrease but rather increased EGFR mRNA levels (Figure 3B). Consistent with our previous study [37], EGFR mRNA levels were reduced upon cell detachment (Figure 3B), but si-FAM188B treatment upregulated EGFR mRNA levels even in suspension conditions. However, FAM188B knockdown decreased EGFR protein levels (Figure 3A). Proteasomal inhibitor, MG132 attenuated EGFR downregulation induced by si-FAM88B treatment (Figure 3C). These data indicate that FAM188B does not regulate EGFR at the transcriptional level but rather through affecting protein stability. FAM188B possibly regulates EGFR ubiquitination status directly or indirectly to modulate EGFR stability, which remains to be determined further. We previously demonstrated that NOX4 upregulation is important for anoikis resistance via maintenance of EGFR levels and activity. NOX4 knockdown decreased both EGFR levels and activity, leading to anoikis sensitivity [37]. We tested whether FAM188B regulates EGFR levels via NOX4. Consistent with the previous report [37], cell detachment upregulated NOX4, but si-FAM188B treatment did not downregulate NOX4 levels (Figure S5), indicating that EGFR downregulation is independent of NOX4 in FAM188B-knockdown cells.

Along with EGFR downregulation, FAM188B knockdown resulted in the inactivation of various signaling pathways that are downstream from EGFR, such as Akt, Src, and STAT3 (Figure 3D). The Akt, Src, and STAT3 pathways are known to be important for anoikis resistance. For example, anoikis-resistant pancreatic cancer cells exhibited significantly increased expression and activation of STAT3 [21]. Src activation is a well-known pathway for anoikis resistance [38], and IQGAP1 upregulation in hepatitis B virus-positive hepatocellular carcinoma cells promotes anoikis resistance, migration, and invasion via activation of Src/FAK signaling [39]. FAM188B knockdown did not affect total levels of Akt, Src, and STAT3 (Figure S6) but decreased their activities (Figure 3D), indicating that FAM188B might affect the upstream regulator(s) of those molecules. Interestingly, unlike Akt, Src, and STAT3, FAK protein levels were downregulated (Figure S6), which might lead to a decreased FAK activity induced by si-FAM188B treatment. It is possible that EGFR downregulation induced by si-FAM188B results in inactivation of those signaling pathways, which are involved not only in the determination of anoikis resistance but also metastatic characteristics, including migration and invasion [40]. FAM188B knockdown attenuated invasion, migration, adhesion, and wound closure (Figure 4A–D). In addition, FAM188B knockdown decreased cell growth in the 3D culture condition, which mimics in vivo conditions (Figure 4E). FAM188B knockdown decreased levels of Survivin, which is an anti-apoptotic protein that plays a critical role in anoikis resistance [14]. Recently, we reported that FAM188B knockdown decreased levels of FOXM1, an oncogenic transcription factor, with an increase in its ubiquitination in A549 cells [14]. Since Survivin expression can be regulated by both STAT3 and FOXM1 [24,25], Survivin downregulation by si-FAM188B treatment is probably due to STAT3 inactivation and/or FOXM1 downregulation (Figure 3D).

Finally, we examined whether FAM188B regulates tumor metastasis in vivo. Injection of the si-FAM188B-treated A549 cells via the tail vein of nude mice resulted in a decrease in lung metastasis and a longer survival when compared with si-NC-treated A549 cells (Figure 5A–E). Furthermore, FAM188B overexpression in the Beas-2B normal lung cells resulted in a little increase in cell aggregation in the suspension culture (Figure S7). All these data indicate that FAM188B regulates anoikis resistance via signaling pathways that are involved in anoikis resistance and metastatic processes, leading to tumor metastasis and poor survival. How FAM188B directly or indirectly regulates levels of oncogenic proteins, including EGFR and FOXM1, remains to be further elucidated. Nonetheless, our results suggest that targeting FAM188B is a potential strategy to prevent cancer metastasis.

## 4. Materials and Methods

### 4.1. Materials

We purchased anti-FAM188B (AbFrontier, Seoul, Korea), anti-p-EGFR (44-788G) (Invitrogen, San Jose, CA, USA), and anti-p-FAK (611722) (BD Bioscience, San Jose, CA, USA). Anti-GAPDH (5174S), anti-p-STAT3 (9145S), anti-p-Akt (4060L), anti-p-Src (2101S), anti-Survivin (2808S), and anti-Ki-67 (9027) were purchased from Cell Signaling Technology (Beverly, MA, USA). Anti-EGFR (sc-03) and anti-FOXM1 (sc-271746) were purchased from Santa Cruz Biotechnology (Santa Cruz, CA, USA). Anti-Vinculin (ab129002) was purchased from Abcam (Cambridge, UK). Horseradish peroxidase (HRP)-conjugated rabbit IgG and HRP-conjugated mouse IgG were purchased from Enzo Life Sciences (Farmingdale, NY, USA). Calcein AM (C3099) was purchased from Invitrogen (Carlsbad, CA, USA). Propidium iodide solution (P4684) and poly (2-hydroxyethyl methacrylate) (Poly-HEMA) were purchased from Sigma-Aldrich Corporation (St. Louis, MO, USA).

### 4.2. Cell Culture

Human lung cancer cell lines, A549 and H1975, were obtained from the American Type Culture Collection (ATCC, Rockville, MD, USA). Human lung cancer cell line H1299 and the normal human lung epithelial cell line Beas-2B were kindly gifted by Dr. Kyungsil Yoon (National Cancer Center, Gyeonggi-do, Korea) and Yeul Hong Kim (Korea University, Seoul, Korea), respectively. A549, H1299, and H1975 cells were grown in RPMI-1640 (Hyclone, Logan, UT, USA) supplemented with 10% FBS (Hyclone, Logan, UT, USA), 100 units/mL penicillin, and 100 μg/mL streptomycin (Gibco Laboratories Co., Grand Island, NY, USA) at 37 °C in a humidified atmosphere containing 5% CO_2_. Beas-2B cells were grown in Keratinocyte-SFM (Gibco, Waltham, MA, USA) containing 0.1 ng/mL epidermal growth factor (EGF) and 15 μg/mL bovine pituitary extract (BPE).

### 4.3. Suspension Culture

Tissue culture plates (60 mm) were coated with 400 μL of poly-HEMA (50 mg/mL in 95% ethanol) and dried overnight in a laminar flow at room temperature. Cells were trypsinized into a single cell suspension, and 4 × 10^5^ cells were plated on poly-HEMA-coated dishes. After 24 h, cells were harvested by centrifugation and processed for cell viability, flow cytometric analysis, and protein analysis.

### 4.4. Live/Dead Viability Assay

A549, H1299, and H1975 cells were transfected with si-NC and si-FAM188B. After 48 h, cells were plated on poly-HEMA coated 60 mm plates in suspended cultures (3 × 10^5^ cells/3 mL) for 24 h. Live or dead cells were analyzed by live/dead viability assay. Briefly, cells were stained with 1 μM calcein AM and 1 μM propidium iodide (PI) for 30 min at 37 °C in a humidified atmosphere containing 5% CO_2_. The labeled cells with two-color fluorescence (green-live cells, red-dead cells) were analyzed by an Axio Observer Z1 fluorescence microscope (Carl Zeiss Microimaging, Thornwood, NY, USA) and an Axion Vision camera (Axion Technologies, Houston, TX, USA).

### 4.5. Small Interfering RNA Preparation and Transfection

Negative control with scrambled sequence was provided by Bioneer (Daejeon, Korea) and small interfering RNA (si-RNA) duplex of human FAM188B was provided by Qiagen (Hilden, Germany; SI04333014). The FAM188B target sequence was 5′-CTG ACC ATT GAC ACC ACC CAA-3′. For RNA interference, A549, H1299, and H1975 cells were transfected with 10 nM si-RNA using Lipofectamine RNAiMAX reagent (Invitrogen, Carlsbad, CA, USA) by reverse transfection according to the manufacturer’s guidelines. After 24 h or 48 h transfection, A549, H1299, and H1975 cells were cultured in either cell culture plates or poly-HEMA-coated plates. Knockdown efficiency and specificity of each si-RNA were confirmed by using immunoblotting with corresponded antibodies.

### 4.6. Immunoblot Analysis

After washing with ice-cold PBS, cells were lysed with 2X SDS lysis buffer (20 mM Tris, 2 mM EDTA, 1 mM Na_3_VO_4_, 2 mM DTT, 2% SDS and 20% glycerol) and boiled for 5 min. The protein concentration of each sample was determined by using the microBCA protein assay reagent (Thermo scientific, Rockford, IL, USA). Five micrograms of total cellular protein was separated by 8% or 10% SDS-PAGE and transferred to PVDF membrane. The membranes were blocked for 60 min at room temperature in tris-buffered saline and tween 20 (TBS-T) containing 5% non-fat dried milk. The membranes were incubated with the primary antibody overnight at 4 °C, washed three times with TBS-T for 30 min, incubated with HRP-conjugated goat anti-mouse IgG or goat anti-rabbit IgG secondary antibodies for 1 h at room temperature, and then washed with TBS-T three times for 30 min. The labeled proteins were visualized by the enhanced chemiluminescence method. The levels of protein were quantified by a densitometry and normalized to loading control GAPDH or Vinculin. Detailed information about immunoblots can be found in Supplemental Data (Figure S8).

### 4.7. Wound Healing Assay

Cells were transfected with si-NC, si-FAM188B for 24 h. Cells were seeded in 12-well plate and cultured to 80–90% confluence. Cells were scratched with sterile tips, and images were captured using a phase-contrast microscopy every hour for 24 h after wounding.

### 4.8. Cell Adhesion and Cell Proliferation Assay

Cells were transfected with si-NC, si-FAM188B for 24 h. For cell adhesion assay, cells were seeded to 96-well culture plate to 7 × 10^3^/well. And then, we removed culture media after 1 h, 2 h, and 4 h. We measured cells that remained in culture plates. For cell proliferation assay, cells transfected with si-NC, si-FAM188B for 24 h and then plated at 5 × 10^3^/well in a 96-well plate. Cell growth was determined with MTS after 24 h and 48 h. Cells were determined using the CellTiter 96 Kit (MTS,3-(4,5-dimethylthiazol-2-yl)-5-(3-carboxyme-thoxyphenyl)-2-(4-sulfophenyl)-2H-tetrazolium; Promega, Madison, WI, USA) as previously described.

### 4.9. Soft Agar Colony Forming Assay

Cells were transfected with siRNAs for 24 h. Soft agar assays were performed in a 6-well plate by placing 5 × 10^3^ cells in 1 mL of 0.45% agar onto a 2 mL of 0.9% agar base layer. Plates were covered with 1 mL fresh RPMI medium containing 10% FBS and incubated in a 5% CO_2_ atmosphere at 37 °C for two weeks. Cell growth medium was changed every third day. Colonies were stained with iodonitrotetrazolium violet (INT) solution (0.5 mg/mL, Sigma), and images were taken with Kodak Image Station 2000R (Eastman Kodak Company, New Haven, CT, USA). Each experiment was conducted in triplicate.

### 4.10. Transwell Migration and Invasion Assay

For migration assay, cells were transfected with si-NC or si-FAM188B for 24 h, and then 1.5 × 10^5^ cells/well were cultured in the Transwell upper chamber (8 μm pore, Corning, Corning, NY, USA). The lower chamber was filled with RPMI-1640 supplemented with 10% FBS. Following 16 h incubation, the lower chamber was isolated, and cells were fixed using 10% formaldehyde at room temperature (RT) for 10 min. Cells were then stained with 0.5% crystal violet. Results represented the mean of the area in three different areas under a light microscope (magnification, ×100). For invasion assays, the upper chambers were pre-coated with 80 μL of Matrigel (1:10 dilution in serum-free RPMI medium), and cells were cultured in the upper chambers for 24 h. Protocols were similar, as described in the migration assay.

### 4.11. Quantitative Real-Time PCR

Total RNA was extracted from the samples with Ribospin (GeneAll, Lisbon, Portugal). One microgram of total RNA was converted into cDNA by using Maxime™ RT PreMix (iNtRON, Burlington, VT, USA), and quantitative RT-PCR analysis was performed on the Roche Light Cycler^®^ 96 by SYBR-GREEN qPCR method (Roche, Basel, Switzerland). All reactions were performed in triplicate, and the relative transcript abundance of each tested gene was normalized to the expression level of the housekeeping gene. cDNA fragments were amplified using the following primer pairs: human EGFR 5′-ACT GCT GCC ACA ACC AGT G-3′ (sense), 5′-GGC TTC GTC TCG GAA TTT G-3′ (anti-sense), human GAPDH 5′-TCT CTG CTC CTC CTG TTC-3′ (sense), 5′-CGC CCA ATA CGA CCA AAT-3′ (anti-sense). Error bars represent the standard deviation (SD) of the mean of triplicate measurements.

### 4.12. Colony Forming Assay

A549, H1299, and H1975 cells were transfected with si-NC, si-FAM188B for 24 h, and cells (4 × 10^2^) were plated in a 6-well plate in 2 mL fresh RPMI medium containing 10% FBS. After two weeks, colonies were fixed with 4% formaldehyde and stained with 5% crystal violet solution, and images were taken by Kodak Image Station 2000R (Eastman Kodak Company, New Haven, CT, USA).

### 4.13. Flow Cytometry Analysis

For the apoptosis assay, cells with si-NC and si-FAM188B were harvested and incubated for 15 min at RT with FITC-conjugated annexin V reagent (2.5 μg/mL) and propidium iodide (PI) (5 μg/mL) in binding buffer followed by flow cytometry analysis. The data analysis was performed using Cell Quest software (BD Biosciences, San Jose, CA, USA).

### 4.14. 3D Culture Assay

A549, H1299, and H1975 cells were transfected with si-NC, si-FAM188B for 24 h, and 3D culture assays were performed in eight well chamber slides (Nunc™ Lab-Tek™, Thermo Fisher Scientific, Waltham, MA, USA) by placing 5 × 10^3^ cells in 400 μL of 5% Matrigel onto a base layer 100 μL of 100% Matrigel. The plates were then incubated in a 5% CO_2_ atmosphere at 37 °C for nine days. Images were taken by using an Axio Observer Z1 fluorescence microscope (Carl Zeiss Microimaging, Thornwood, NY, USA) and an Axion Vision camera (Axion Technologies, Houston, TX, USA).

### 4.15. Lung Metastasis in Vivo and Immunohistochemical Staining of Tumor Tissues

A549 cells transfected with si-NC and si-FAM188B and cells (1 × 10^6^/100 μL) were injected into the tail vein of BALB/c nude mice (*n* = 5 in each group). The mice were sacrificed after eight weeks, lungs were harvested, and the number of tumor nodules in the lungs was counted. Lungs were fixed in 4% paraformaldehyde and stained with hematoxylin-eosin staining solution (Sigma, St. Louis, MO, USA). The tumor tissues incubated with primary antibodies for 1 h were then treated with anti-rabbit biotinylated antibody (1:1000 dilutions; Vector Laboratories, San Francisco, CA, USA) for 1 h. Color reaction was developed by incubation with diaminobenzidine solution (Sigma) followed by counterstaining with hematoxylin. Stained tissues were reviewed by two experienced pathologists. This study was reviewed and approved by the Institutional Animal Care and Use Committee (IACUC) of the National Cancer Center Research Institute (NCCRI), and IACUC approval number is NCC-16-231. NCCRI is an Association for Assessment and Accreditation of Laboratory Animal Care International (AAALAC International) accredited facility and abides by the Institute of Laboratory Animal Resources (ILAR) guide.

### 4.16. Statistical Analysis

For survival data, the statistical analysis was performed by use of GraphPad Prism 8 (GraphPad Software, San Diego, CA, USA). Kaplan–Meier curves were plotted and compared using a log-rank test. Comparisons between two groups were performed using a Student’s *t*-test. Statistical significance was defined as * *p* < 0.05 and ** *p* < 0.01. Data represent average values and standard deviations (error bars) obtained from three independent experiments.

## 5. Conclusions

In conclusion, FAM188B expression is critical for tumor malignancies, such as anoikis resistance and tumor metastasis. How FAM188B directly or indirectly regulates levels of oncogenic proteins, including EGFR and FOXM1, remains to be further elucidated. Nonetheless, our results suggest that targeting FAM188B is a potential strategy to prevent cancer metastasis.

## Data Availability

No new data were created or analyzed in this study. Data sharing is not applicable to this article.

## Supplementary Materials

The following are available online at https://www.mdpi.com/2072-6694/13/2/247/s1, Figure S1: Effects of FAM188B knockdown on apoptosis of lung cancer cells, Figure S2: Effects of FAM188B knockdown on cell aggregation upon cell detachment, Figure S3: Effects of FAM188B overexpression on the EGFR levels in the FAM188B-knockdown and densitometry analysis of immunoblot in Figure 3, Figure S4: Effects of FAM188B knockdown on lung metastasis and STAT3 activation in xenograft mouse model, Figure S5: Effects of FAM188B knockdown on NOX4 expression, Figure S6: Effects of FAM188B knockdown on levels of STAT3, Akt, Src, and FAK, Figure S7: Effects of FAM188B overexpression on the cell aggregate formation in the Beas-2B cells, Figure S8: Original Western blots.
