# Supplementary material for: FAM188B Downregulation Sensitizes Lung Cancer Cells to Anoikis via EGFR Downregulation and Inhibits Tumor Metastasis In Vivo
Source: Cancers (Basel). 2021 Jan 11;13(2):247. doi: 10.3390/cancers13020247 (PMC7826942; doi:10.3390/cancers13020247)
Supplement: Supplementary file 1 [file cancers-13-00247-s001.pdf]

cancers

MDPI

Article

FAM188B Downregulation Sensitizes Lung Cancer Cells to Anoikis via EGFR Downregulation and Inhibits Tumor Metastasis in Vivo

Eun-Ju Jang, Jee Young Sung, Ha-Eun Yoo, Hyonchol Jang, Jaegal Shim, Eok-Soo Oh, Sung-Ho Goh and Yong-Nyun Kim

Supplementary materials

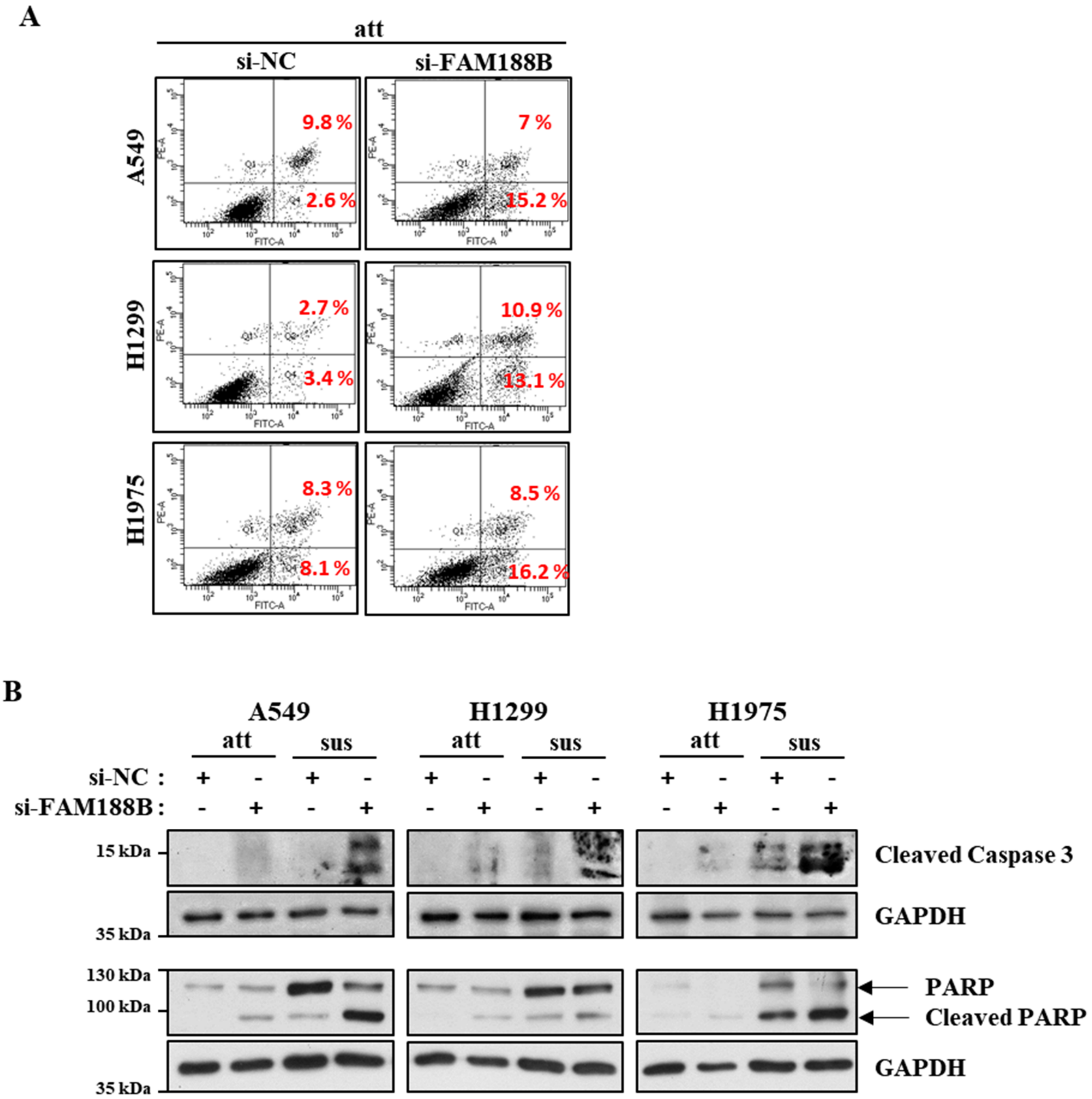

Figure S1. Effects of FAM188B knockdown on apoptosis of lung cancer cells (**A**) A549, H1299, and H1975 cells were transiently transfected with non-specific control si-RNA (si-NC) or si-RNA targeting FAM188B (si-FAM188B) for 48 h. Cells were stained with annexin V/PI, followed by flow cytometry analysis. Apoptotic cells were quantified with early and late apoptotic cell populations. (**B**) Cells were transfected as in (**A**) for 48 h, and cells were then further cultured in

either attached (att) or suspended (sus) condition for 24 h, followed by immunoblot analysis using indicated antibodies. GAPDH was used as a loading control. These experiments were performed three times independently with similar results.

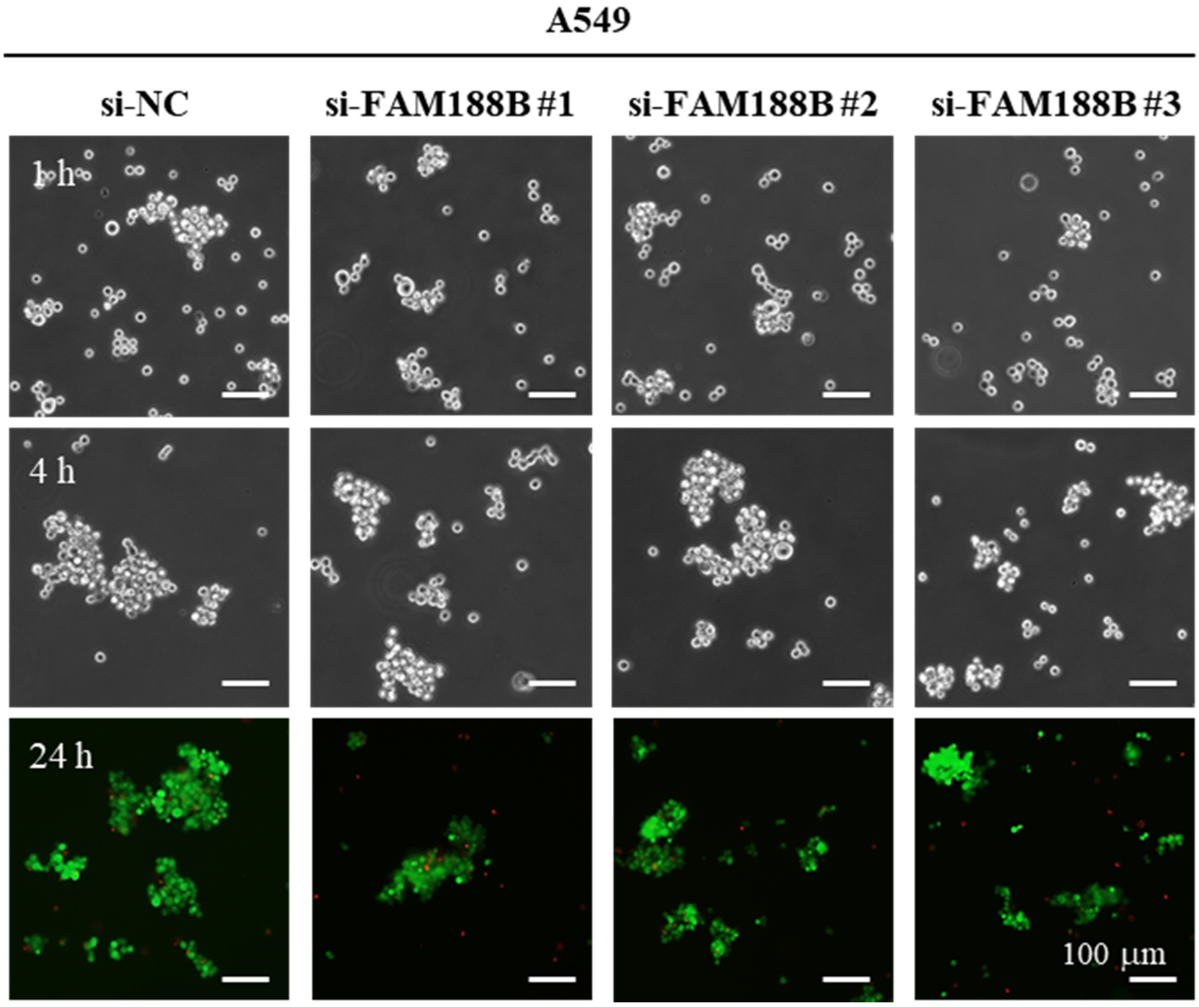

Figure S2. Effects of FAM188B knockdown on cell aggregation upon cell detachment A549 Cells were transiently transfected with si-NC or three different si-RNAs targeting FAM188B (si-FAM188B #1, #2, and #3) for 24 h. Cells were then grown in the HEMA-coated plate for suspension culture for 24 h, followed by calcein-AM/PI staining for live/dead cell assay. Cell images were taken at the indicated times. Scale bars = 100 µm. These experiments were performed two times independently with similar results.

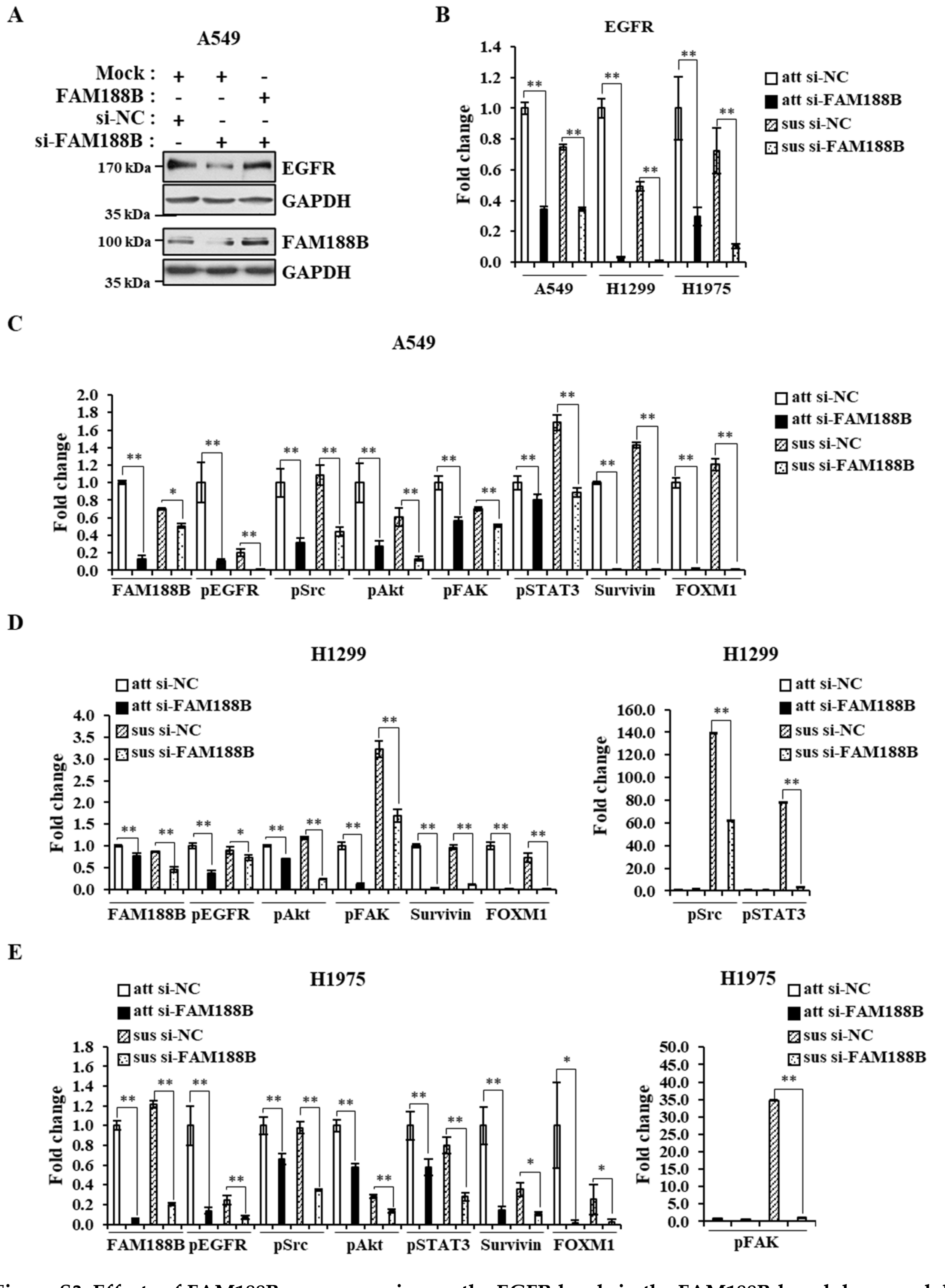

Figure S3. Effects of FAM188B overexpression on the EGFR levels in the FAM188B-knockdown and densitometry analysis of immunoblot in Figure 3 (**A**) A549 cells were transfected with si-NC or si-FAM188B in the absence (Mock) or presence of FAM188B overexpression (FAM188B). Cells were then processed for immunoblot analysis using indicated antibodies. GAPDH was used as a loading control. (**B**–**E**) Densitometry analysis of immunoblot in Figure 3. A549, H1299, and H1975 cells were transiently transfected with non-specific control si-RNA (si-NC) or si-RNA targeting FAM188B (si-FAM188B) for 48 h. The histogram shows the densitometric measuring of EGFR expression relative to GAPDH (Figure 3A) (B). The histogram shows the densitometric measuring of FAM188B, pEGFR (Y1068), pAkt (S473), pSrc (Y416), pFAK (Y397), pSTAT3 (Y705), Survivin, and FOXM1 expression relative to GAPDH (Figure 3D). The histogram shows the densitometric measuring of Survivn expression relative to Vinculin in H1299 cells (Figure 3D) (**C**–**E**). Error bars represent standard deviations of the mean of three measurements (*$p < 0.05$, **$p < 0.01$, *versus* si-NC). These experiments were performed two times independently with similar results.

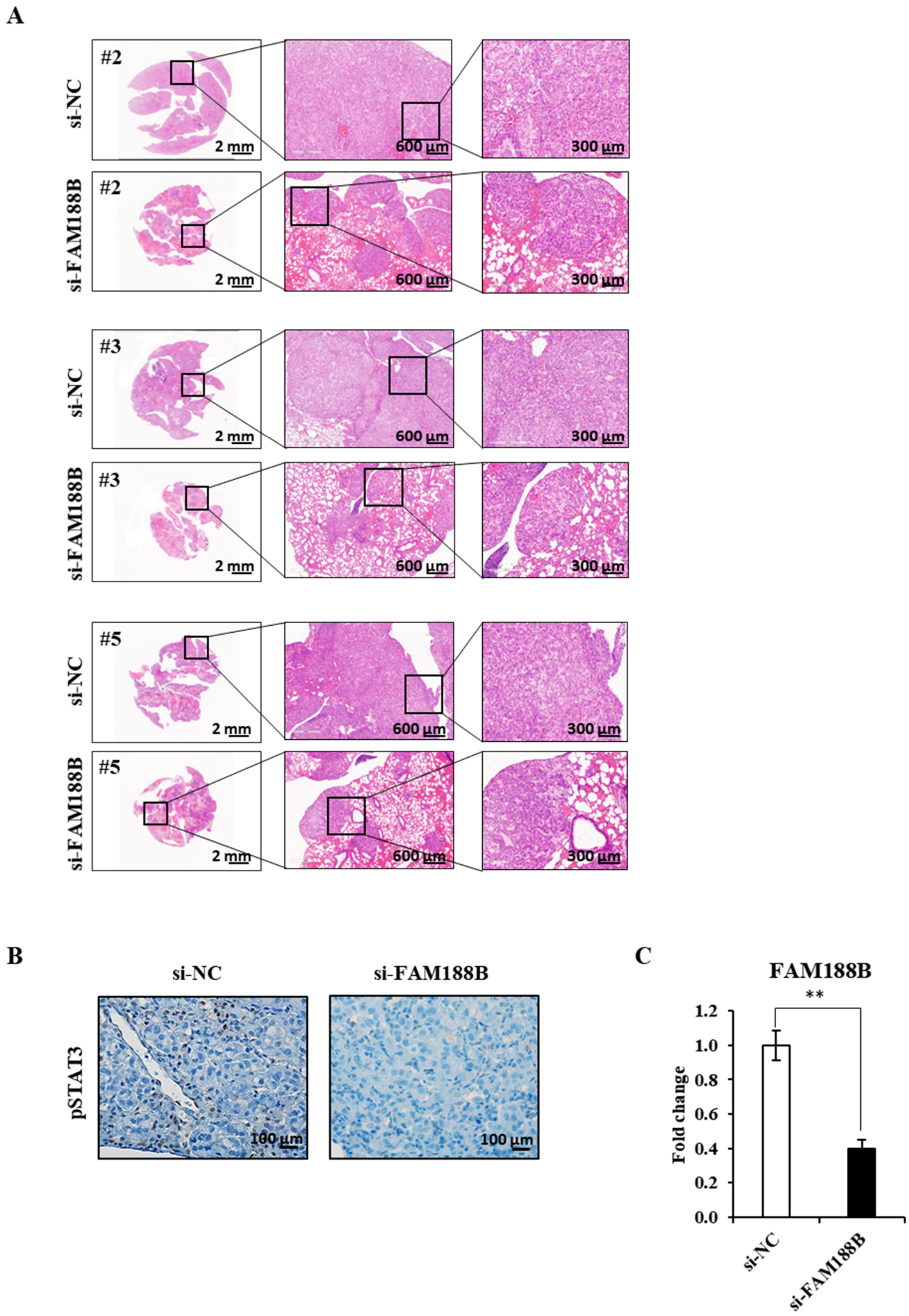

Figure S4. Effects of FAM188B knockdown on lung metastasis and STAT3 activation in xenograft mouse model (**A**–**C**) A549 were transiently transfected with either si-NC or si-FAM188B for 48 h and cells were injected into BALB/c nude mice (n = 10) via tail vein. Mice were sacrificed after 8 weeks and lung tissues were stained with H&E (**A**) or with anti-pSTAT3 (Y705) antibodies (**B**). Scale bars = 2 mm, 600 μm, and 300 μm (**A**), 100 μm (**B**). The histogram shows the densitometric measuring of FAM188B expression relative to GAPDH (Figure 5A) (**C**). Error bars represent standard deviations of the mean of three measurements (**p* < 0.05, ***p* < 0.01, *versus* si-NC). Similar results were observed in two independent experiments.

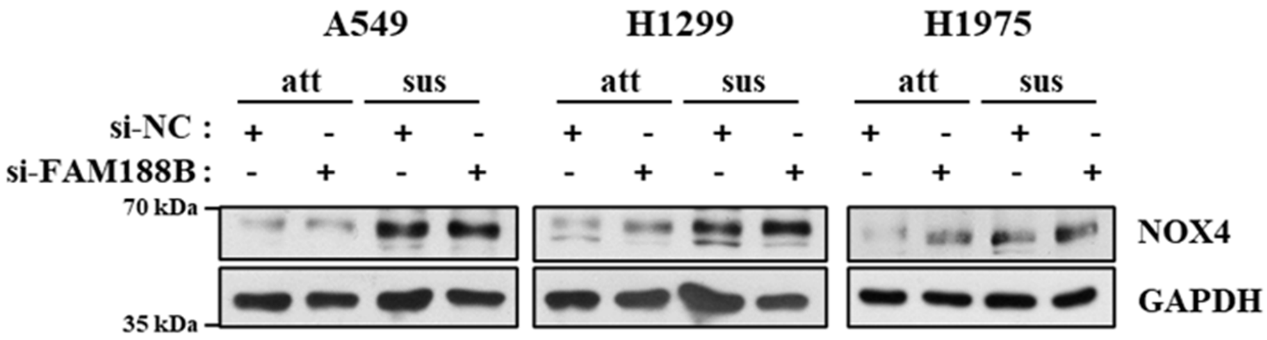

Figure S5. Effects of FAM188B knockdown on NOX4 expression Cells were transiently transfected with either si-NC or si-FAM188B for 48 h, followed by either attached or suspended culture for 24 h. Cells were then processed for immunoblot analysis using indicated antibodies. GAPDH was used as a loading control. These experiments were performed two times independently with similar results.

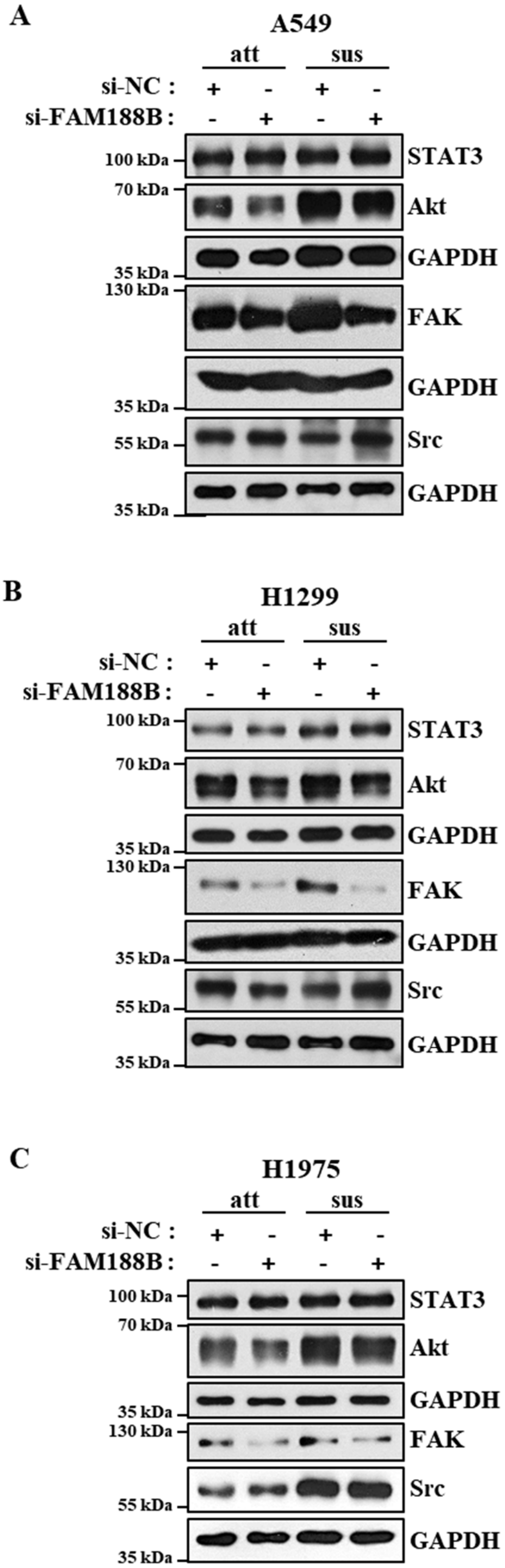

Figure S6. Effects of FAM188B knockdown on levels of STAT3, Akt, Src, and FAK (**A**–**C**) Cells were transiently transfected with either si-NC or si-FAM188B for 48 h, followed by either attached or suspended culture for 24 h. Cells were then processed for immunoblot analysis using indicated antibodies. GAPDH was used as a loading control. These experiments were performed three times independently with comparable results.

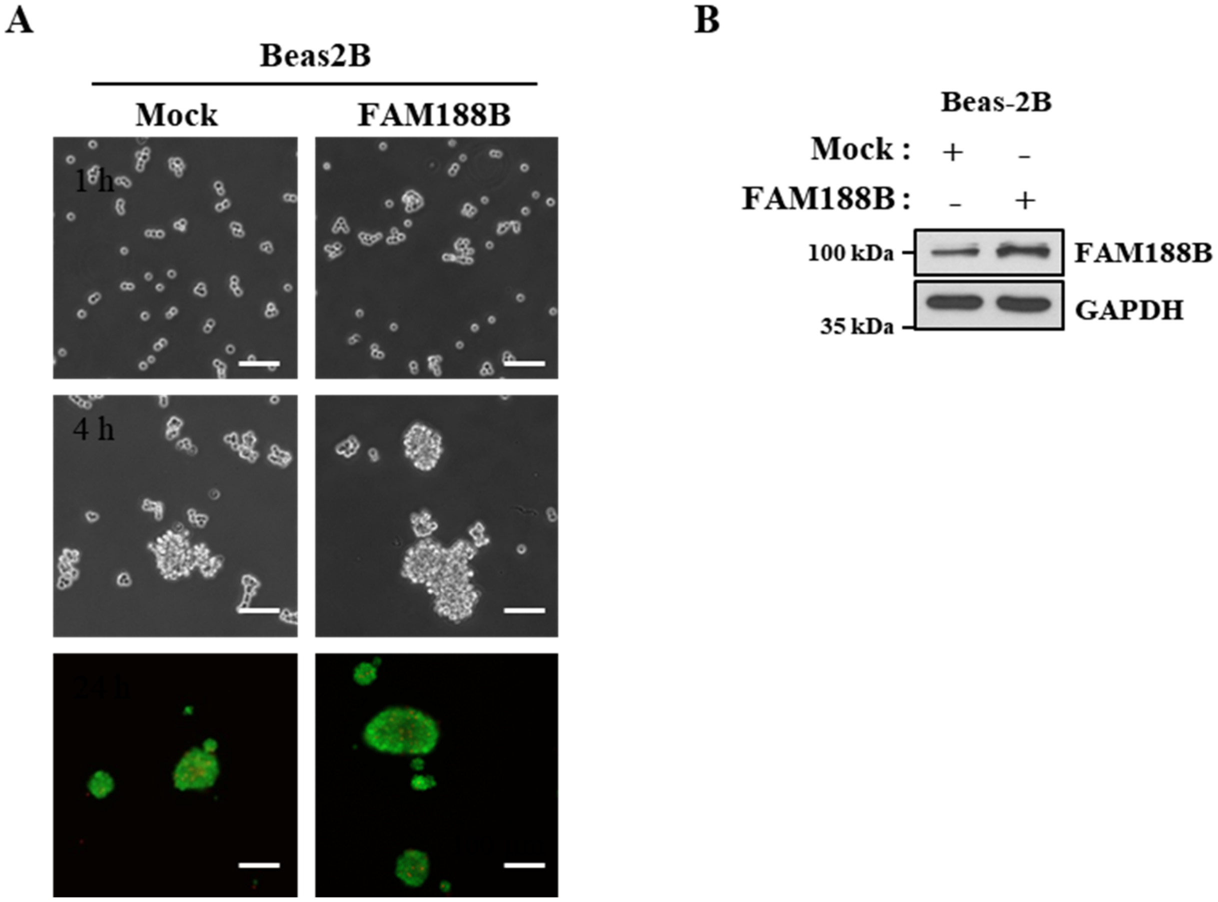

Figure S 7. Effects of FAM188B overexpression on the cell aggregate formation in the Beas-2B cells (**A**–**B**) Human normal lung epithelial cell line, Beas-2B, was transfected with mock and FAM188B for 24 h, and then cells were grown in the HEMA-coated plate for suspension culture for 24 h, followed by calcein-AM/PI staining for live/dead cell assay. Cell images were taken at the indicated times. Scale bars = 100 μm (**A**). Cells were then processed for immunoblot analysis using indicated antibodies. GAPDH was used as a loading control (**B**). These experiments were performed two times independently with similar results.

1. Supplementary Materials and Methods

1.1. Materials

We purchased anti-FAM188B (AbFrontier, Seoul, Korea) and anti-p-EGFR (44-788G) (Invitrogen, San Jose, CA, USA), anti-p-FAK (611722), and anti-FAK (610087) (BD Bioscience, San Jose, CA, USA). Anti-GAPDH (5174S), anti-STAT3 (4904S), anti-p-STAT3 (9145S), anti-Akt (4961S) and anti-Src (2109S), anti-p-STAT3 (9145S), anti-p-Akt (4060L), anti-p-Src (2101S), anti-Survivin (2808S), anti-caspase 3 (9662S), anti-PARP (9542S) were purchased from Cell Signaling Technology (Beverly, MA, USA). NOX4 (ab133303) was purchased from Abcam (Cambridge, UK). Horseradish peroxidase (HRP)-conjugated rabbit IgG and HRP-conjugated mouse IgG were purchased from Enzo Life Sciences (Farmingdale, NY, USA). Calcein AM (C3099) was purchased from Invitrogen (Carlsbad, CA, USA). Propidium iodide solution (P4684) and poly (2-hydroxyethyl methacrylate) (Poly-HEMA) were purchased from Sigma-Aldrich Corporation (St. Louis, MO, USA).

1.2. Cell Culture

Human lung cancer cell lines, A549 and H1975 were obtained from the American Type Culture Collection (ATCC, Rockville, MD). Human lung cancer cell line, H1299 and human normal lung epithelial cell line, Beas-2B were kindly gifted by Dr Kyungsil Yoon (National Cancer Center, Gyeonggi-do, Korea) and Yeul Hong Kim (Korea University,

Seoul, Korea), respectively. A549, H1299, and H1975 cells were grown in RPMI-1640 (Hyclone, Logan, UT, USA) supplemented with 10% FBS (Hyclone, Logan, UT, USA), 100 units/ml penicillin, and 100 μg/ml streptomycin (Gibco Laboratories Co., Grand Island, NY) at 37°C in a humidified atmosphere containing 5% CO_2. Beas-2B cells were grown in Keratinocyte-SFM (Gibco, San Jose, CA, USA) containing 0.1 ng/ml epidermal growth factor (EGF) and 15 μg/ml bovine pituitary extract (BPE).

1.3. Suspension Culture

Tissue culture plates (60 mm) were coated with 400 μl of poly-HEMA (50 mg/ml in 95% ethanol) and dried for overnight in a laminar flow at room temperature. Cells were trypsinized into a single cell suspension and 4×10^5 cells were plated on poly-HEMA-coated dishes. After 24 h, cells were harvested by centrifugation and processed for cell viability, flow cytometric analysis, and protein analysis.

1.4. Flow Cytometry Analysis

For the apoptosis assay, cells with si-NC and si-FAM188B were harvested and incubated for 15 min at RT with FITC-conjugated annexin V reagent (2.5 μg/ml) and propidium iodide (PI) (5 μg/ml) in binding buffer followed by flow cytometry analysis. The data analysis was performed using Cell Quest software (BD Biosciences, San Jose, CA, USA).

1.5. Small Interfering RNA Preparation and Transfection

Negative control with scrambled sequence was provide from Bioneer (Daejeon, Republic of Korea) and validated small interfering RNA (si-RNA) duplex of human FAM188B was provide from Qiagen (Hilden, Germany; SI04333014). The FAM188B target sequence was si-FAM188B #1 (5'-ATA CGG CAT AGT GCA GAA CAA-3'), si-FAM188B #2 (5'-GAG GCT GGA AAG AGC GTT CAA-3'), and si-FAM188B #3 (5'-CTG ACC ATT GAC ACC ACC CAA-3'). A549 cells were transfected with 10 nM si-RNA using Lipofectamine RNAiMAX reagent (Invitrogen, Carlsbad, CA, USA) by reverse transfection according to the manufacturer's guideline. After 24 h transfection, cells were suspended in either cell culture plate or poly-HEMA-coated plates. Knockdown efficiency and specificity of each si-RNA were confirmed by using immunoblotting with corresponded antibodies.

1.6. Live/Dead Viability Assay

A549 cells were transfected with si-NC and si-FAM188B. After 24 h, cells were plated on poly-HEMA coated 60 mm plates in suspended cultures ($3x10^5$ cells/3ml) for 24 h. Live or dead cells were analyzed by live/dead viability assay. Briefly, cells were stained with 1 μM calcein AM and 1 μM propidium iodide (PI) for 30 min at 37°C in a humidified atmosphere containing 5% CO2. The labeled cells with two-color fluorescence (green-live cells, red-dead cells) were analyzed by an Axio Observer Z1 fluorescence microscope (Carl Zeiss Microimaging, Thornwood, NY, USA) and an Axion Vision camera (Axion Technologies, Houston, TX, USA).

1.7. Transfection of Cells

FAM188B/pFLAG-CMV construct was a kind gift from Dr. Sung-Ho Goh (National cancer center, Korea) and was described previously. Beas-2B cells were transfected with FAM188B/pFLAG-CMV or mock vector plasmids using Lipofectamine 3000 reagent (Invitrogen, Carlsbad, CA, USA) for 24 h.

1.8. Immunoblot Analysis

After washing with ice-cold PBS, cells were lysed with 2X SDS lysis buffer (20 mM Tris, 2 mM EDTA, 1 mM Na_3VO_4, 2 mM DTT, 2% SDS and 20% glycerol) and boiled for 5 min. Protein concentration of each sample was determined by microBCA protein assay

reagent (Thermo scientific, Rockford, IL, USA). 5 μg of total cellular protein was separated by 8 or 10% SDS-PAGE and transferred to PVDF membrane. The membranes were blocked for 60 min at room temperature in tris-buffered saline and tween 20 (TBS-T) containing 5% non-fat dried milk. The membranes were incubated with the primary antibody for overnight at 4°C, washed three times with TBS-T for 30 min, incubated with HRP-conjugated goat anti-mouse IgG or goat anti-rabbit IgG secondary antibodies for 1 h at room temperature, and then washed with TBS-T three times for 30 min. The labeled proteins were visualized by the enhanced chemiluminescence method.

1.9. Lung metastasis in Vivo and Immunohistochemical Staining of Tumor Tissues

A549 cells transfected with si-NC and si-FAM188B and cells (1×10^6/100 μl) were injected via tail-vein of BALB/c nude mice (n = 5 in each group). After 8 weeks, the mice were sacrificed, lungs were harvested and fixed in 4% paraformaldehyde and stained with hematoxylin-eosin (H&E) staining solution (Sigma, St. Louis, MO). The tumor tissues incubated with primary antibodies for 1 h were then treated with anti-rabbit biotinylated antibody (1:1000 dilutions; Vector Laboratories) for 1 h. Color reaction was developed by incubation with diaminobenzidine solution (Sigma) followed by counter staining with hematoxylin. Stained tissues were reviewed by two experienced pathologists. This study was reviewed and approved by the Institutional Animal Care and Use Committee (IACUC) of National Cancer Center Research Institute (NCCRI) and IACUC approval number is NCC-16-231. NCCRI is an Association for Assessment and Accreditation of Laboratory Animal Care International (AAALAC International) accredited facility and abides by the Institute of Laboratory Animal Resources (ILAR) guide.

Figure 1A

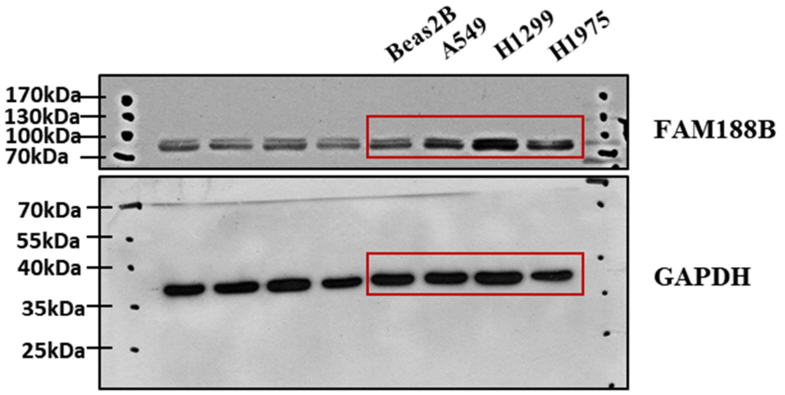

Figure 1B

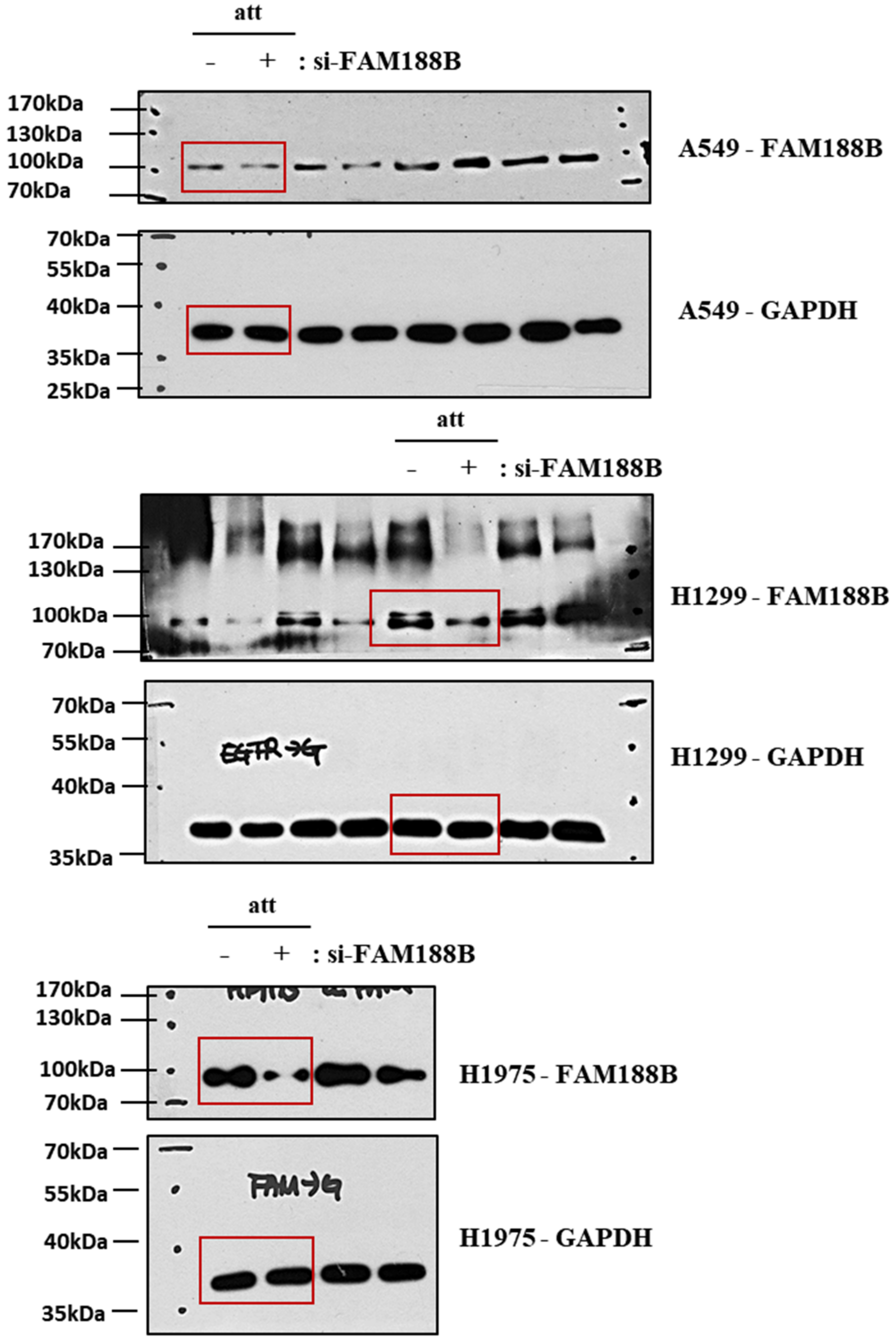

Figure 2A

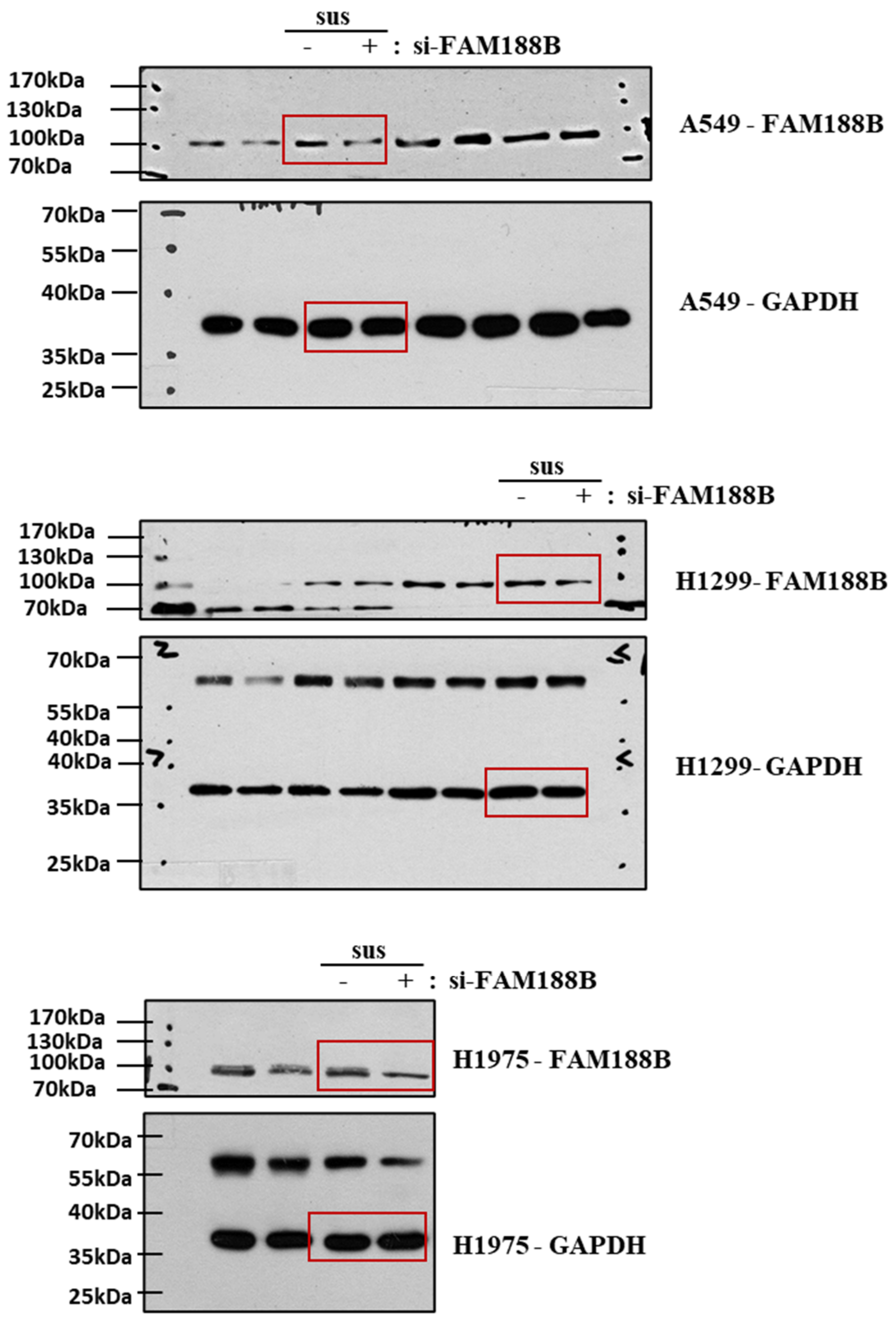
sus
- + : si-FAM188B
170kDa
130kDa
100kDa
70kDa
A549 - FAM188B
70kDa
55kDa
40kDa
35kDa
25kDa
A549 - GAPDH
sus
- + : si-FAM188B
170kDa
130kDa
100kDa
70kDa
H1299- FAM188B
70kDa
55kDa
40kDa
40kDa
35kDa
25kDa
H1299- GAPDH
sus
- + : si-FAM188B
170kDa
130kDa
100kDa
70kDa
H1975 - FAM188B
70kDa
55kDa
40kDa
35kDa
25kDa
H1975 - GAPDH

Figure 3A

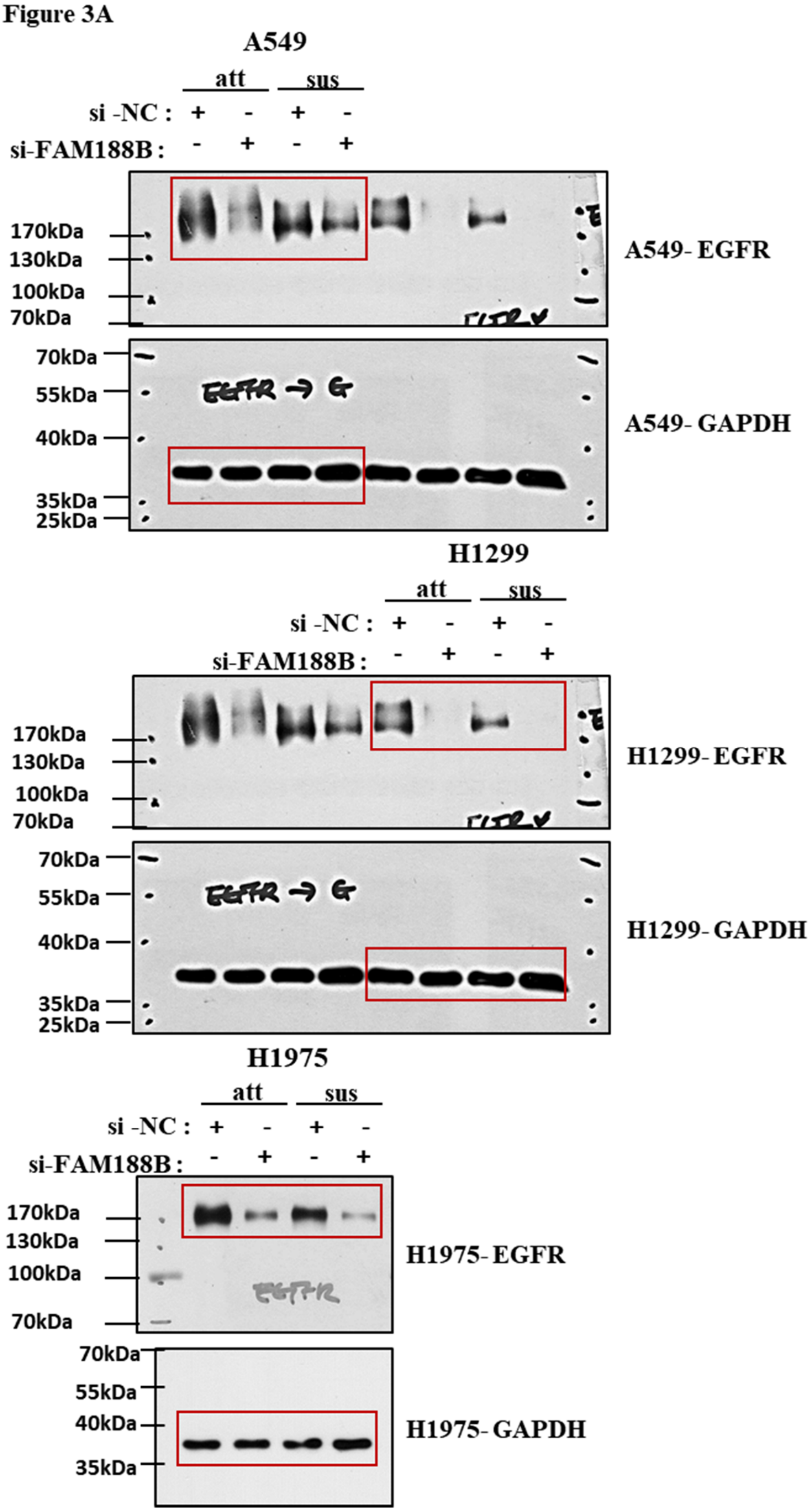
A549
att
sus
si -NC : + - + -
si-FAM188B : - + - +
170kDa
130kDa
100kDa
70kDa
A549- EGFR
70kDa
55kDa
40kDa
35kDa
25kDa
A549- GAPDH
H1299
att
sus
si -NC : + - + -
si-FAM188B : - + - +
170kDa
130kDa
100kDa
70kDa
H1299- EGFR
70kDa
55kDa
40kDa
35kDa
25kDa
H1299- GAPDH
H1975
att
sus
si -NC : + - + -
si-FAM188B : - + - +
170kDa
130kDa
100kDa
70kDa
H1975- EGFR
70kDa
55kDa
40kDa
35kDa
H1975- GAPDH

Figure 3C

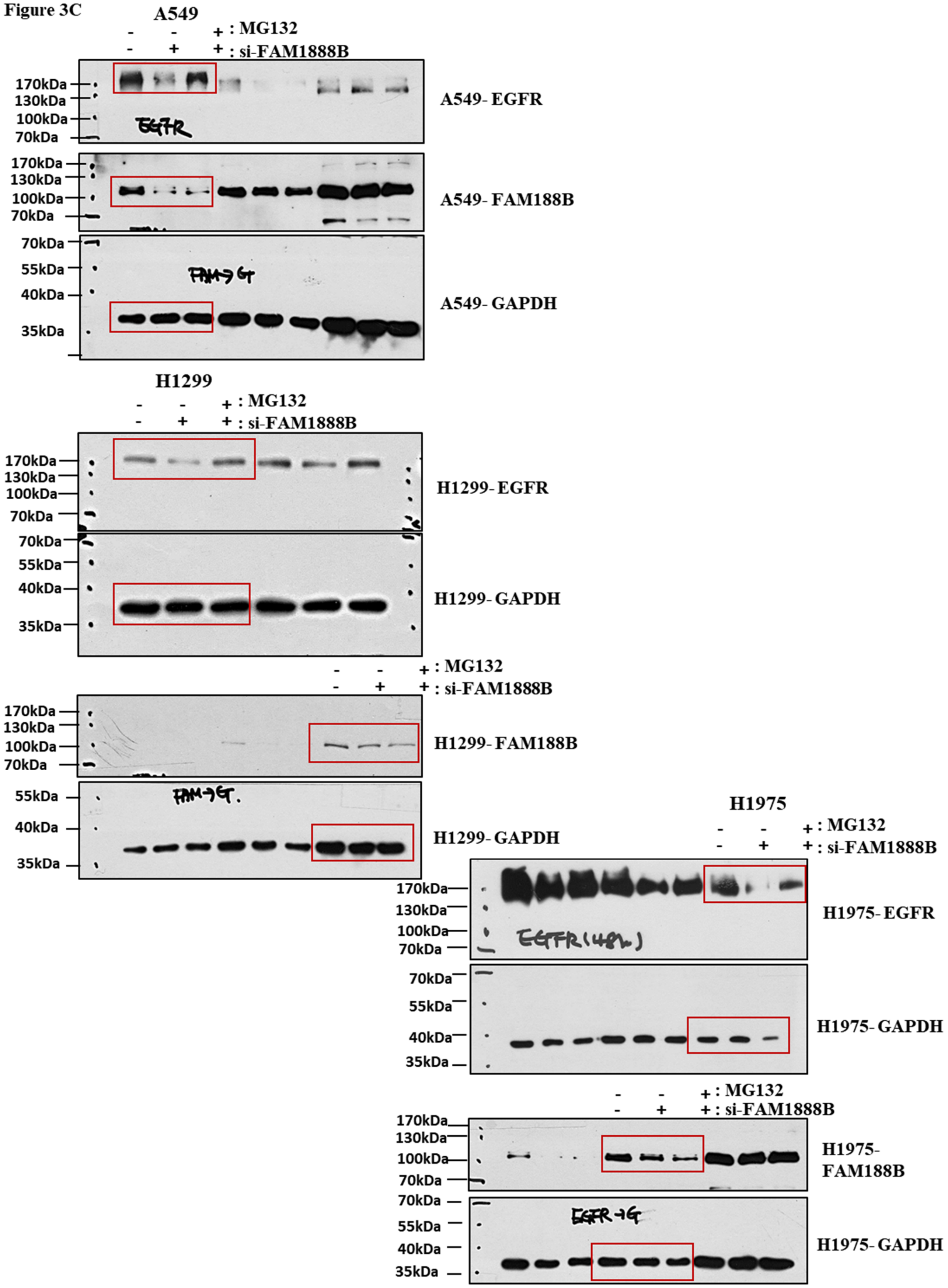
A549
- - + : MG132
- + + : si-FAM1888B
170kDa
130kDa
100kDa
70kDa
A549- EGFR
A549- FAM188B
55kDa
40kDa
35kDa
A549- GAPDH
H1299
H1299- EGFR
H1299- GAPDH
H1299- FAM188B
H1975
H1975- EGFR
H1975- GAPDH
H1975- FAM188B

Figure 3D

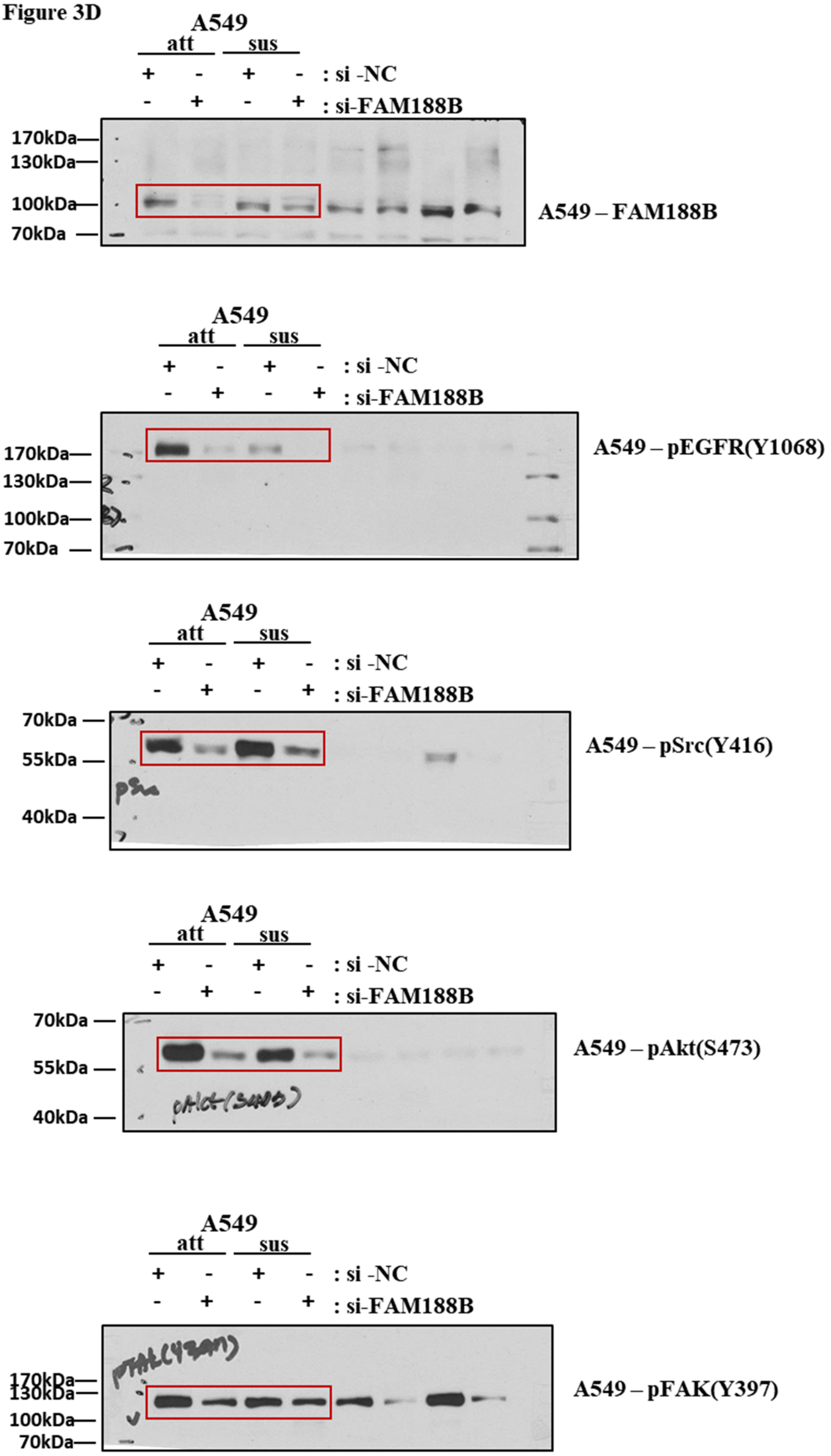
A549
att
sus
+ - + - : si -NC
- + - + : si-FAM188B
170kDa
130kDa
100kDa
70kDa
A549 – FAM188B
A549
att
sus
+ - + - : si -NC
- + - + : si-FAM188B
170kDa
130kDa
100kDa
70kDa
A549 – pEGFR(Y1068)
A549
att
sus
+ - + - : si -NC
- + - + : si-FAM188B
70kDa
55kDa
40kDa
A549 – pSrc(Y416)
A549
att
sus
+ - + - : si -NC
- + - + : si-FAM188B
70kDa
55kDa
40kDa
A549 – pAkt(S473)
A549
att
sus
+ - + - : si -NC
- + - + : si-FAM188B
170kDa
130kDa
100kDa
70kDa
A549 – pFAK(Y397)

Figure 3D

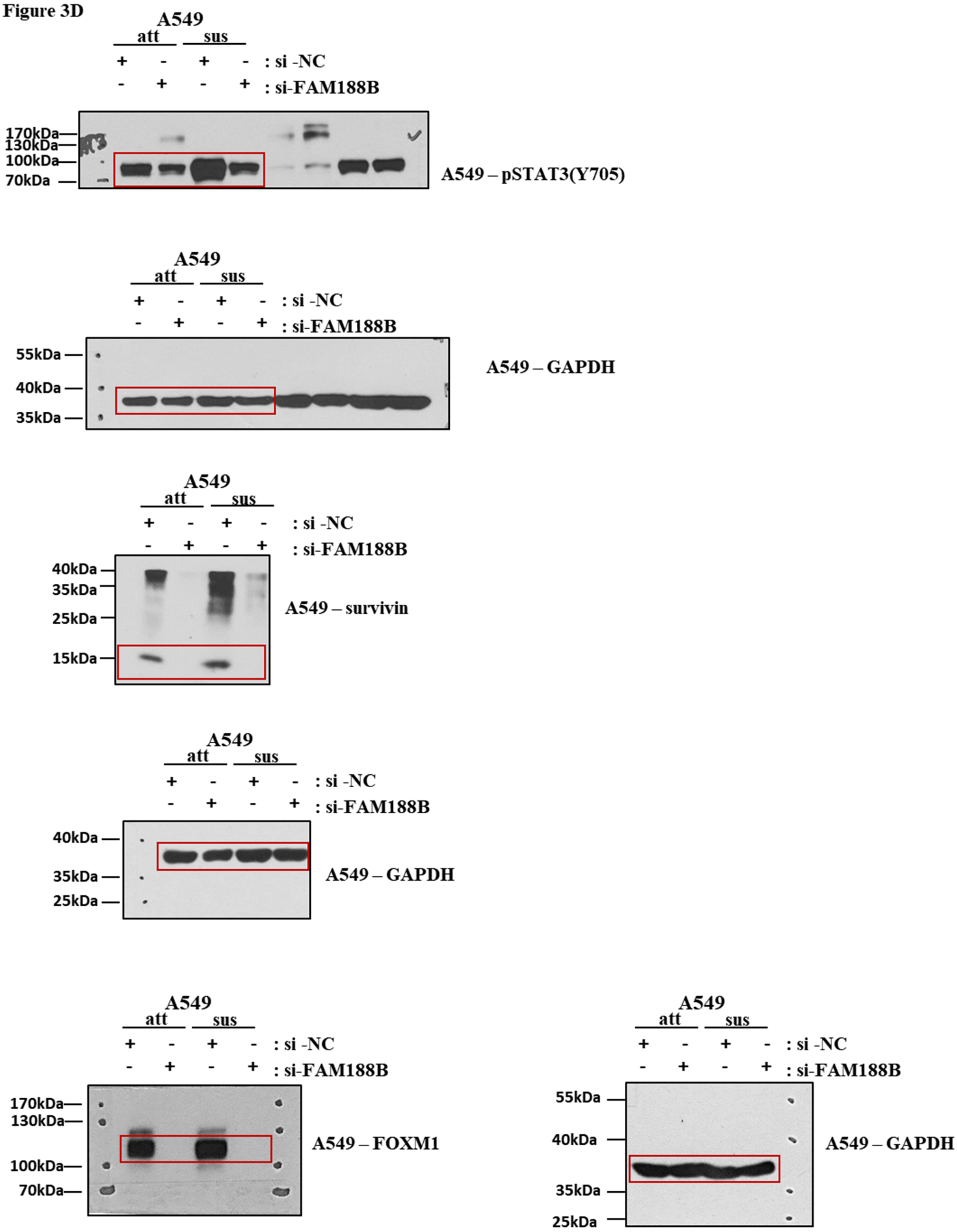
A549
att
sus
: si -NC
: si-FAM188B
170kDa
130kDa
100kDa
70kDa
A549 – pSTAT3(Y705)
A549
att
sus
: si -NC
: si-FAM188B
55kDa
40kDa
35kDa
A549 – GAPDH
A549
att
sus
: si -NC
: si-FAM188B
40kDa
35kDa
25kDa
15kDa
A549 – survivin
A549
att
sus
: si -NC
: si-FAM188B
40kDa
35kDa
25kDa
A549 – GAPDH
A549
att
sus
: si -NC
: si-FAM188B
170kDa
130kDa
100kDa
70kDa
A549 – FOXM1
A549
att
sus
: si -NC
: si-FAM188B
55kDa
40kDa
35kDa
25kDa
A549 – GAPDH

Figure 3D

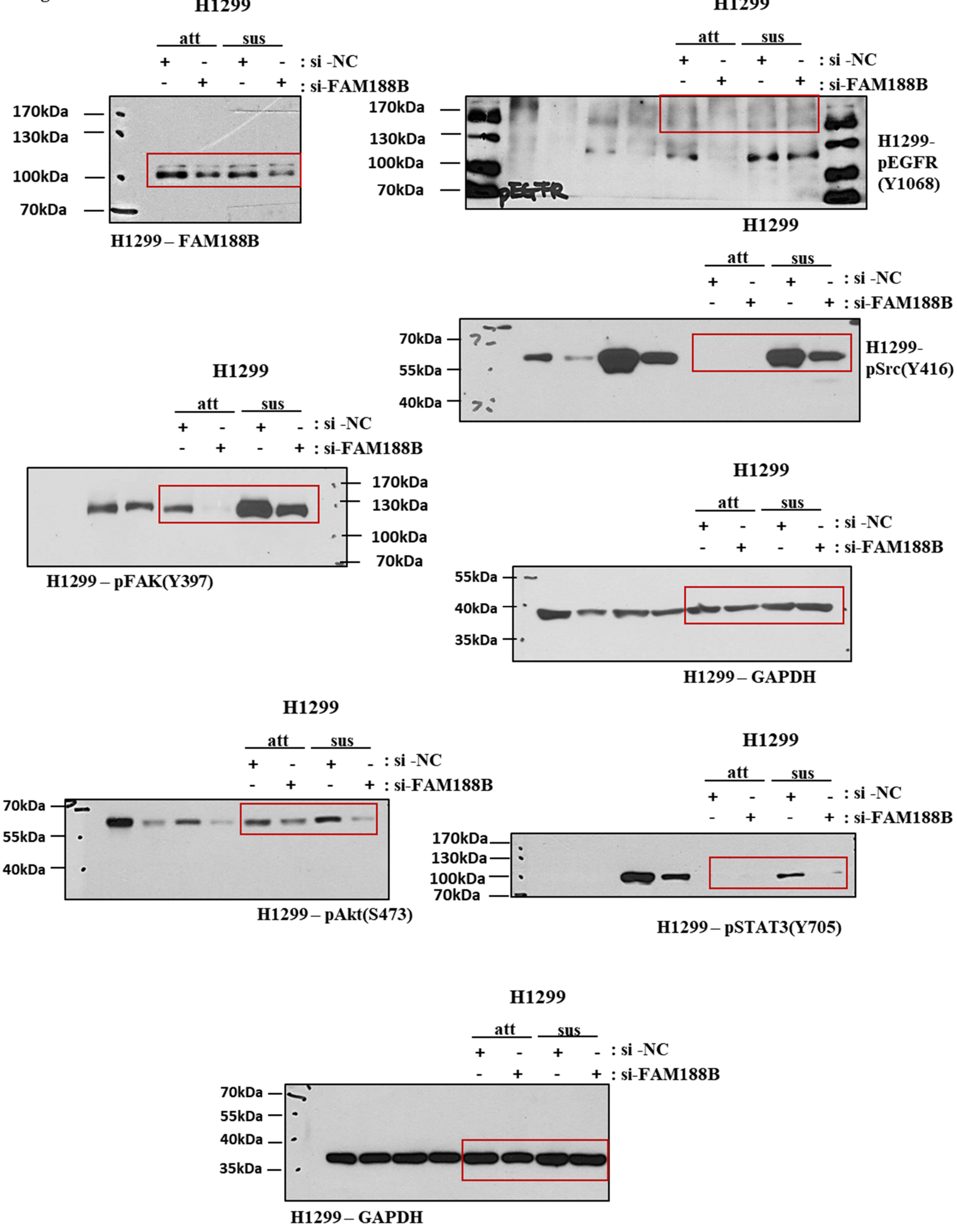
H1299
att
sus
: si -NC
: si-FAM188B
170kDa
130kDa
100kDa
70kDa
H1299 – FAM188B
H1299-
pEGFR
(Y1068)
H1299-
pSrc(Y416)
55kDa
40kDa
H1299 – pFAK(Y397)
35kDa
H1299 – GAPDH
H1299 – pAkt(S473)
H1299 – pSTAT3(Y705)

Figure 3D

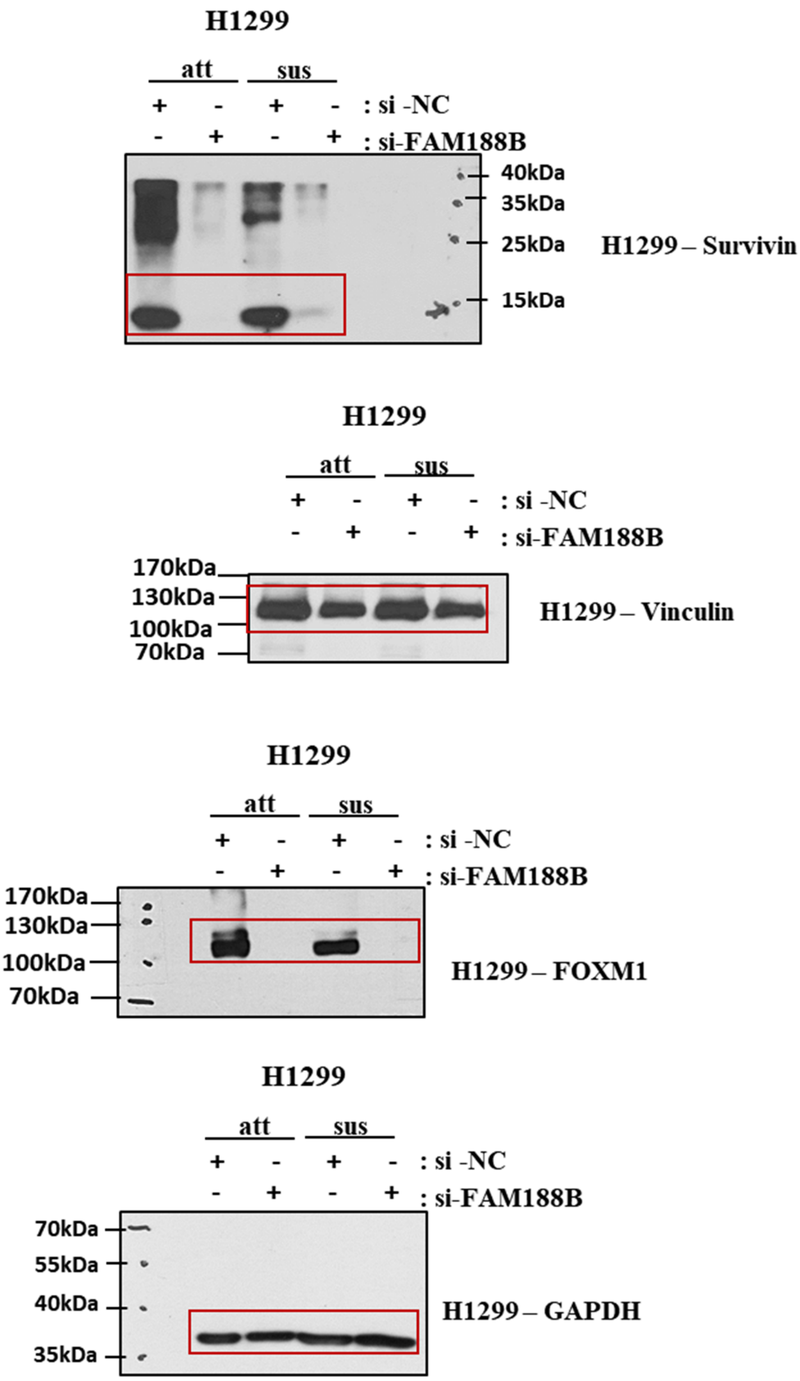
H1299
att
sus
+ - + - : si -NC
- + - + : si-FAM188B
40kDa
35kDa
25kDa
15kDa
H1299 – Survivin
H1299
att
sus
+ - + - : si -NC
- + - + : si-FAM188B
170kDa
130kDa
100kDa
70kDa
H1299 – Vinculin
H1299
att
sus
+ - + - : si -NC
- + - + : si-FAM188B
170kDa
130kDa
100kDa
70kDa
H1299 – FOXM1
H1299
att
sus
+ - + - : si -NC
- + - + : si-FAM188B
70kDa
55kDa
40kDa
35kDa
H1299 – GAPDH

Figure 3D

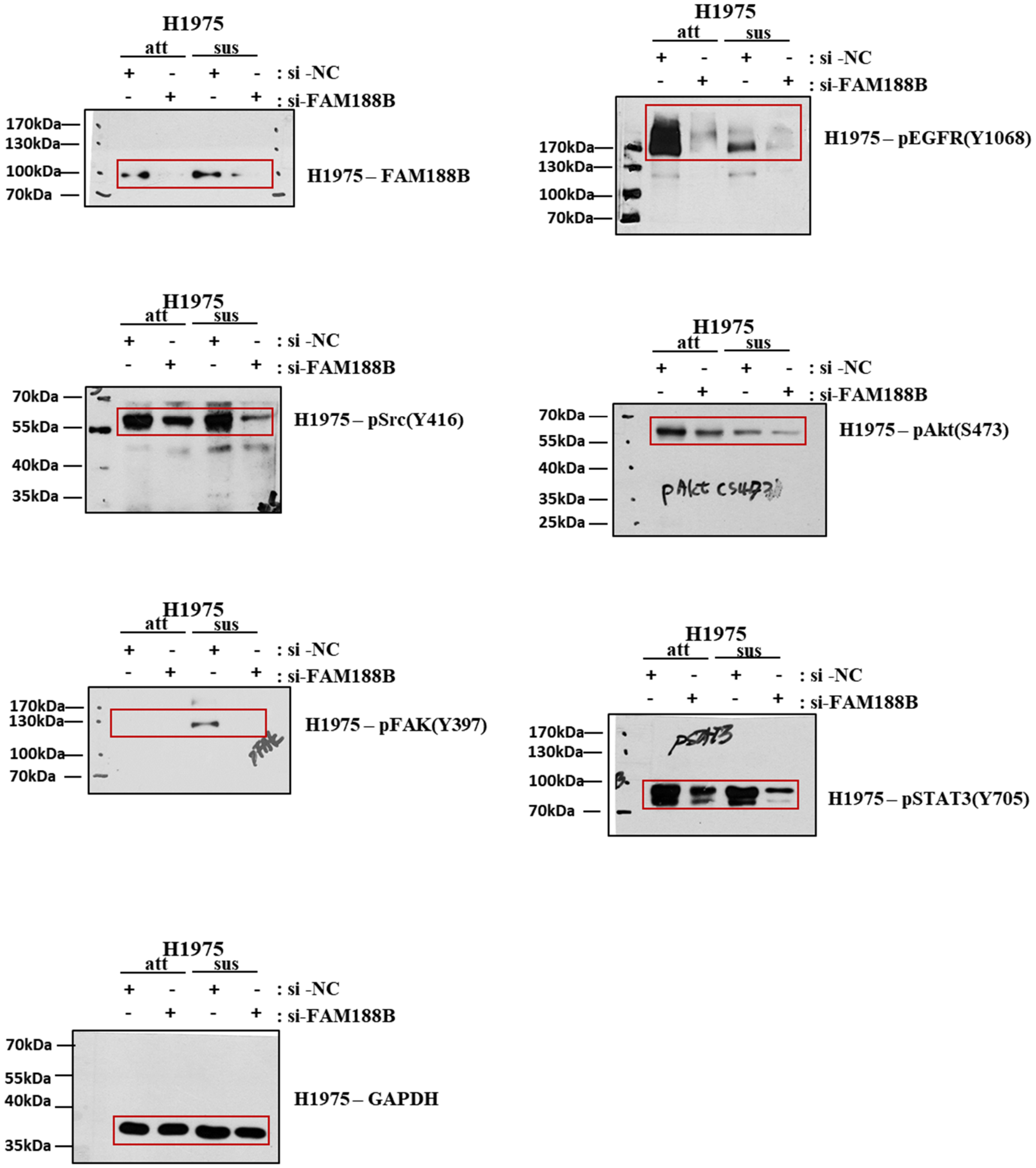

H1975
att
sus
: si -NC
: si-FAM188B
170kDa
130kDa
100kDa
70kDa
H1975 – FAM188B
H1975 – pEGFR(Y1068)
55kDa
40kDa
35kDa
H1975 – pSrc(Y416)
25kDa
H1975 – pAkt(S473)
H1975 – pFAK(Y397)
H1975 – pSTAT3(Y705)
H1975 – GAPDH

Figure 3D

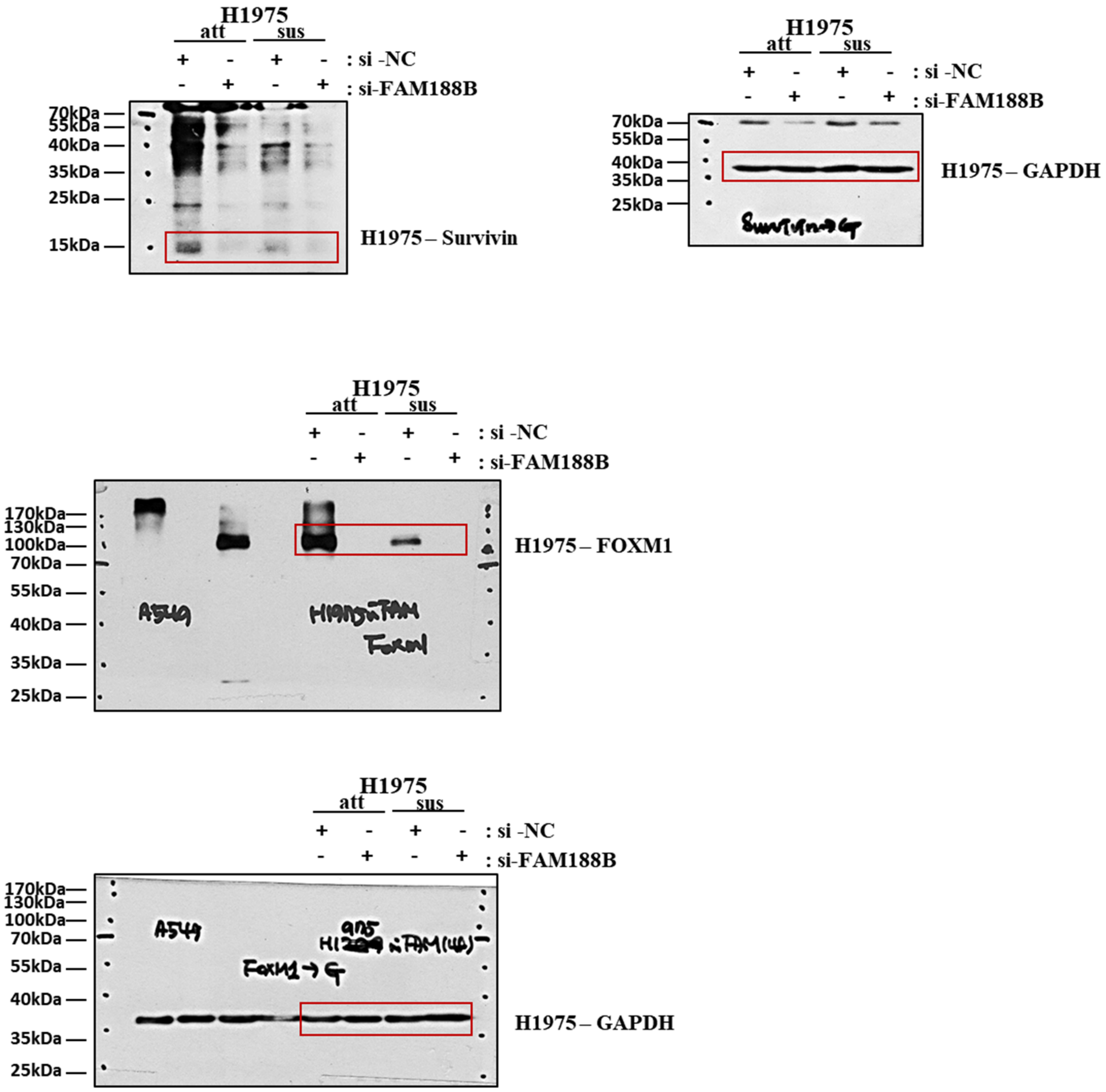
H1975
att
sus
: si -NC
: si-FAM188B
70kDa
55kDa
40kDa
35kDa
25kDa
15kDa
H1975 – Survivin
H1975 – GAPDH
170kDa
130kDa
100kDa
H1975 – FOXM1
A549
H1975 – GAPDH

Figure 5A

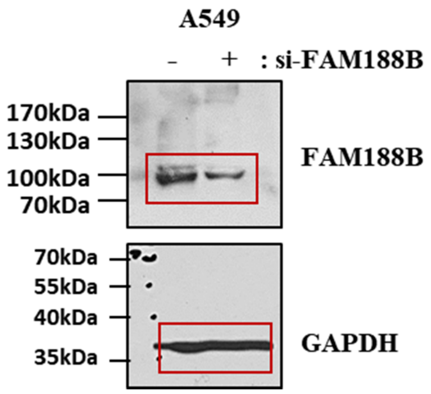
A549
- + : si-FAM188B
170kDa
130kDa
100kDa
70kDa
FAM188B
70kDa
55kDa
40kDa
35kDa
GAPDH

Supplementary figure 1 B

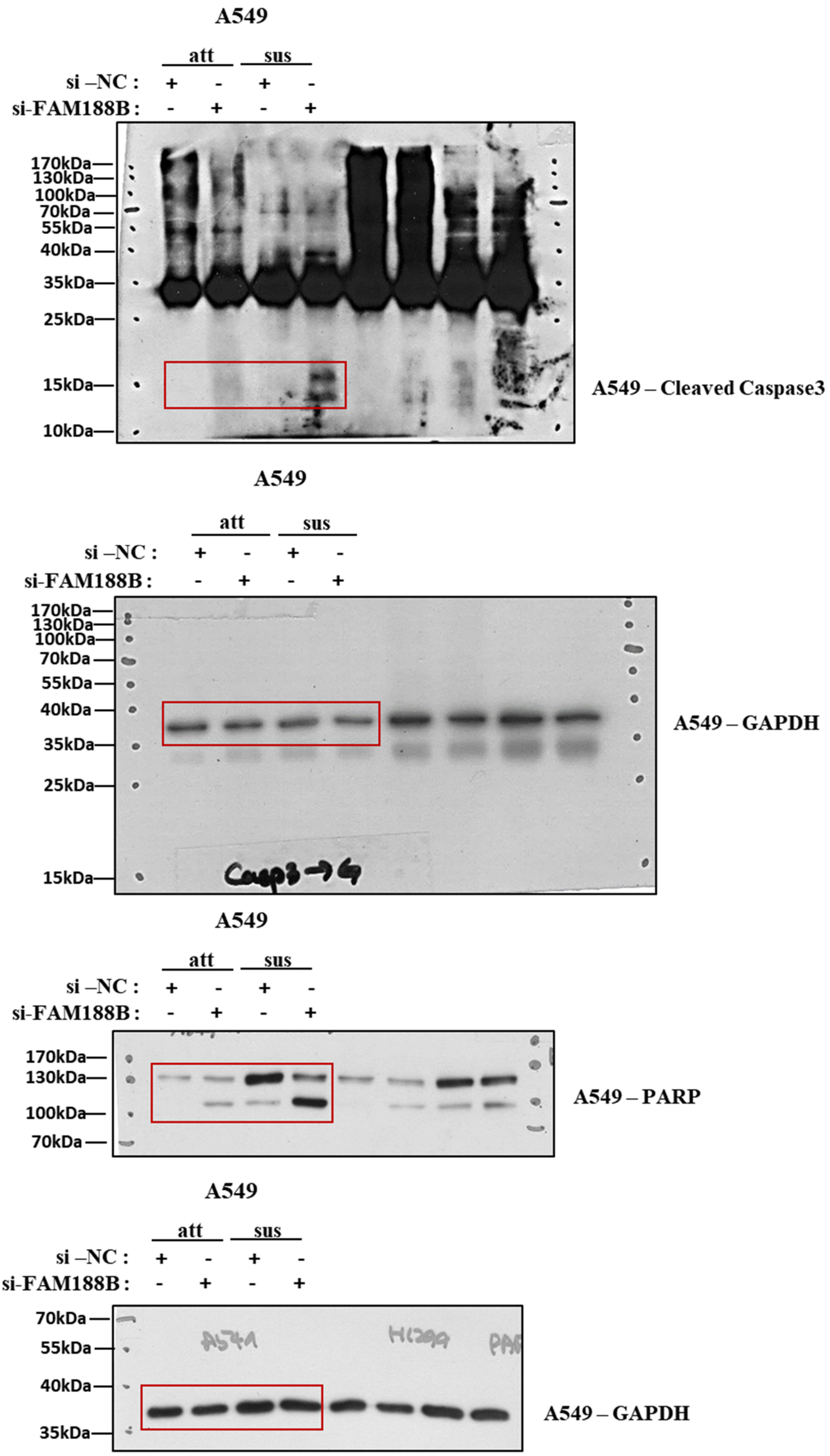

Supplementary figure 1 B

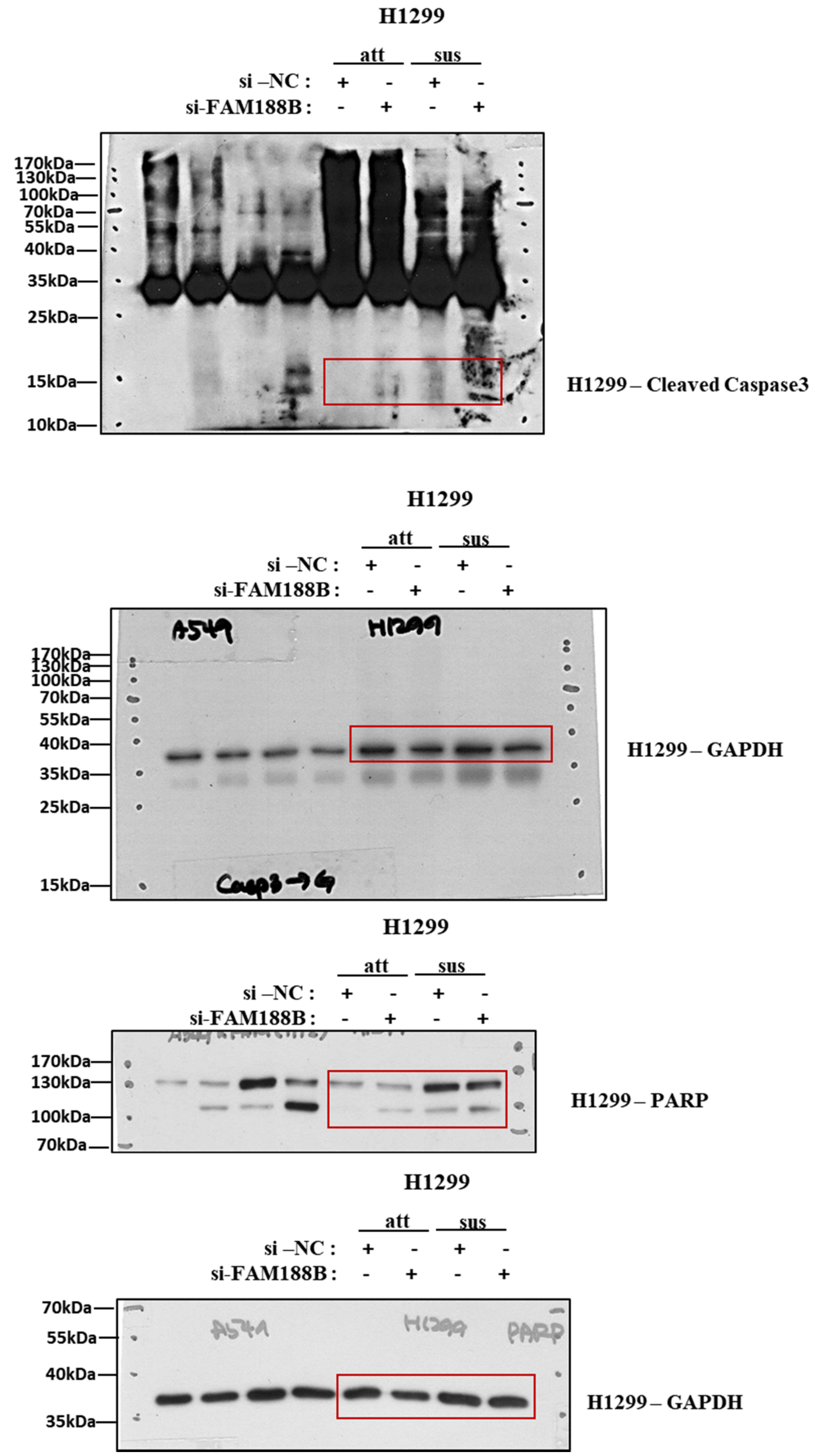

Supplementary figure 1 B

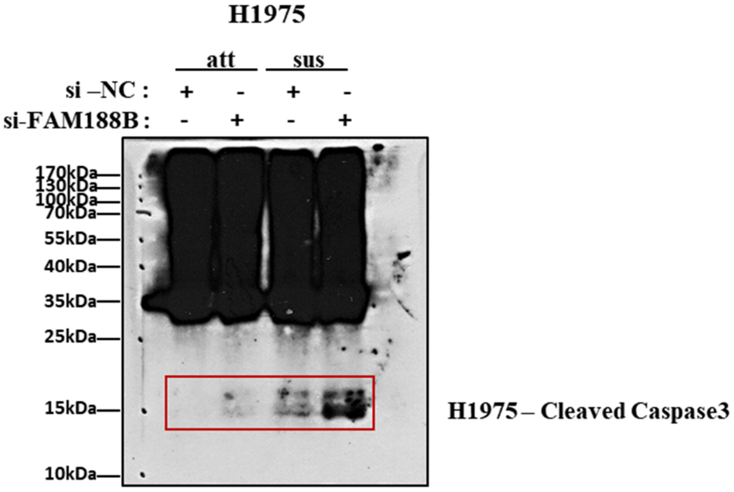

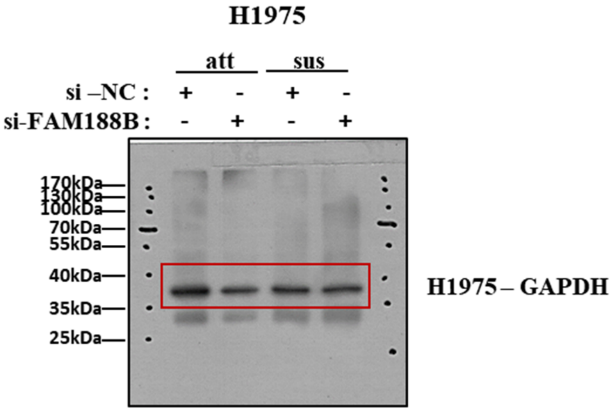

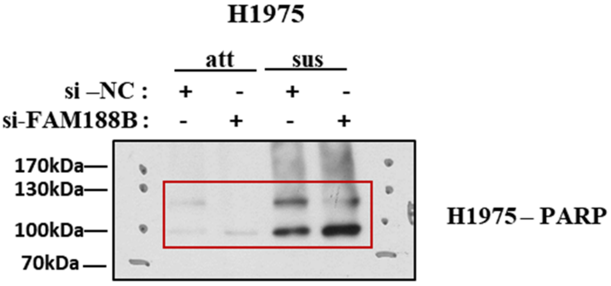

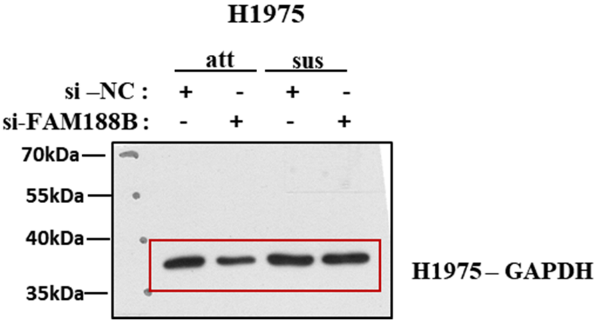

Supplementary figure 3A

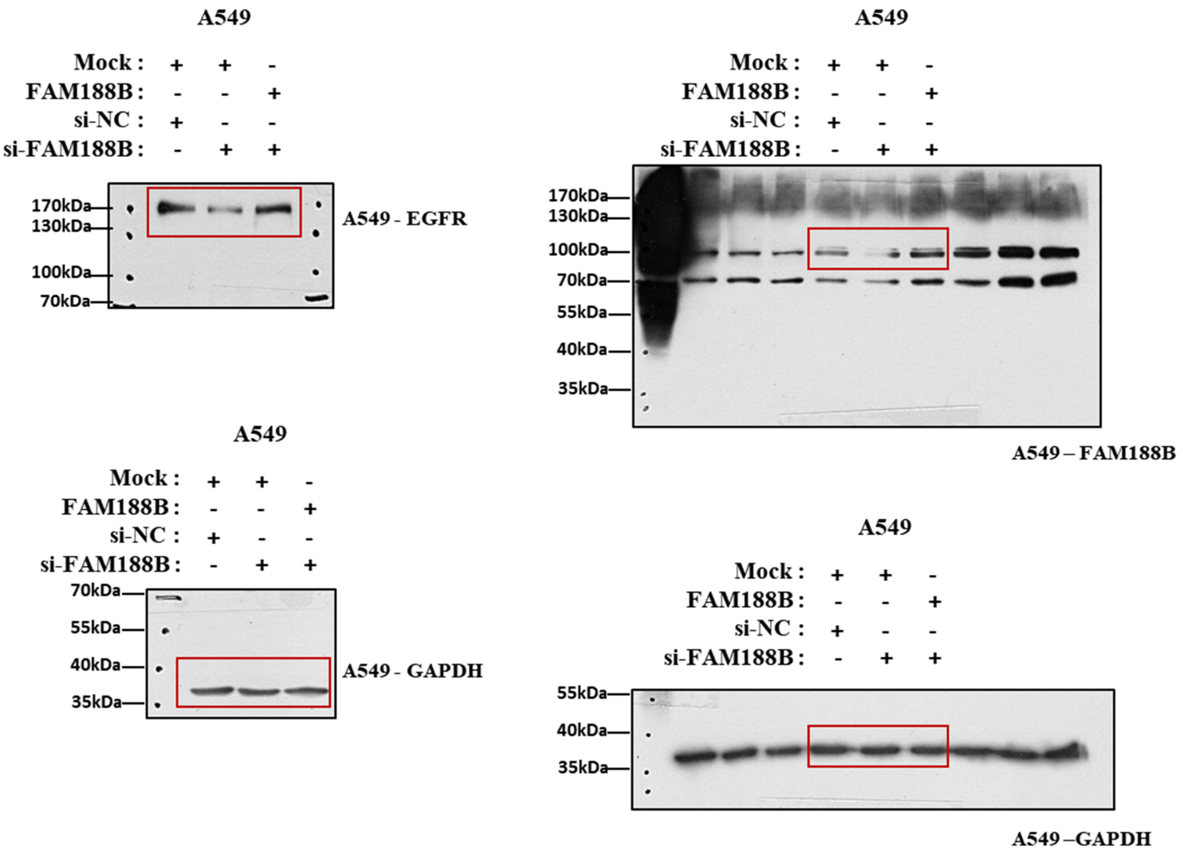

Supplementary figure 5

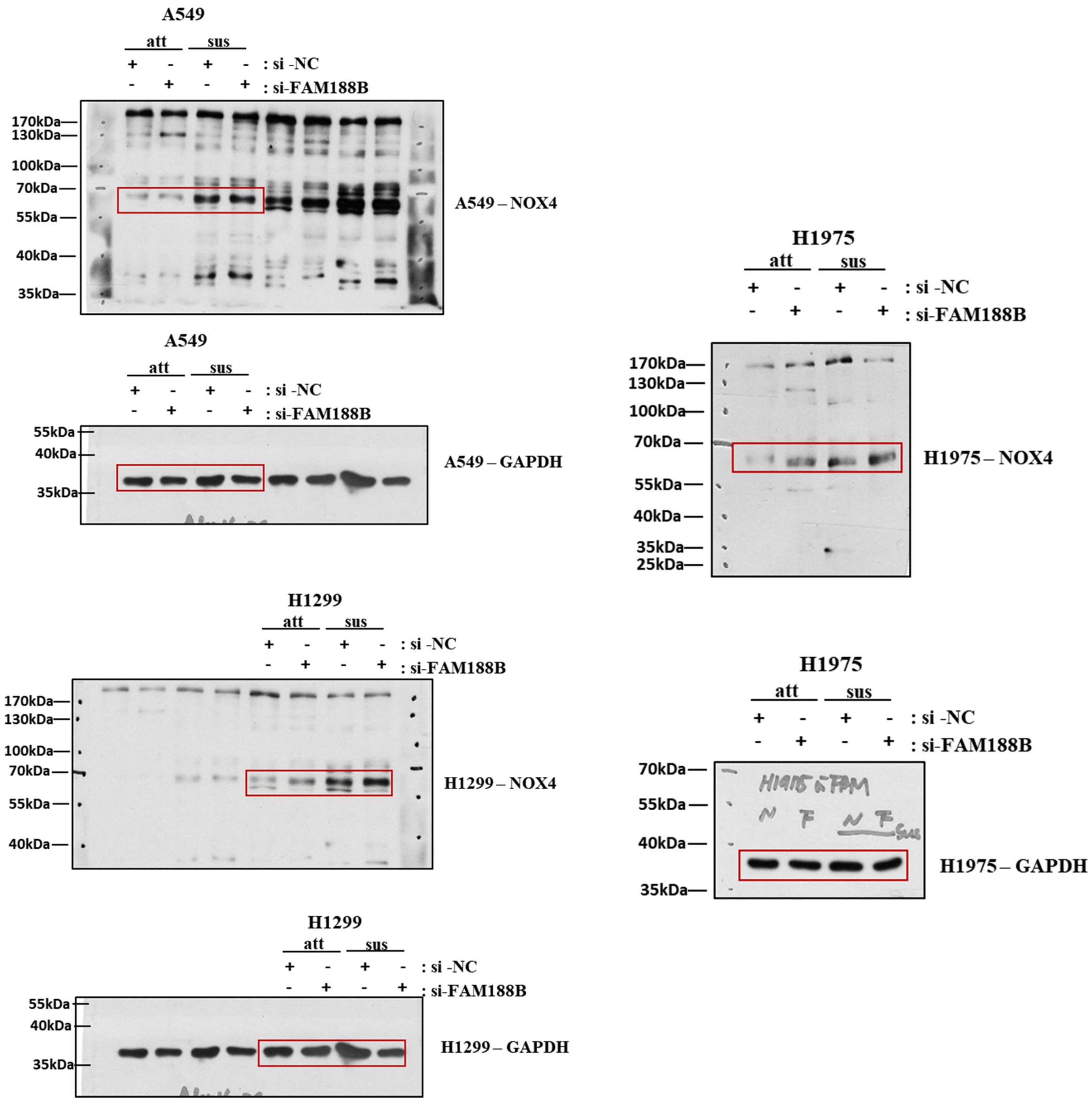

Supplementary figure 6A

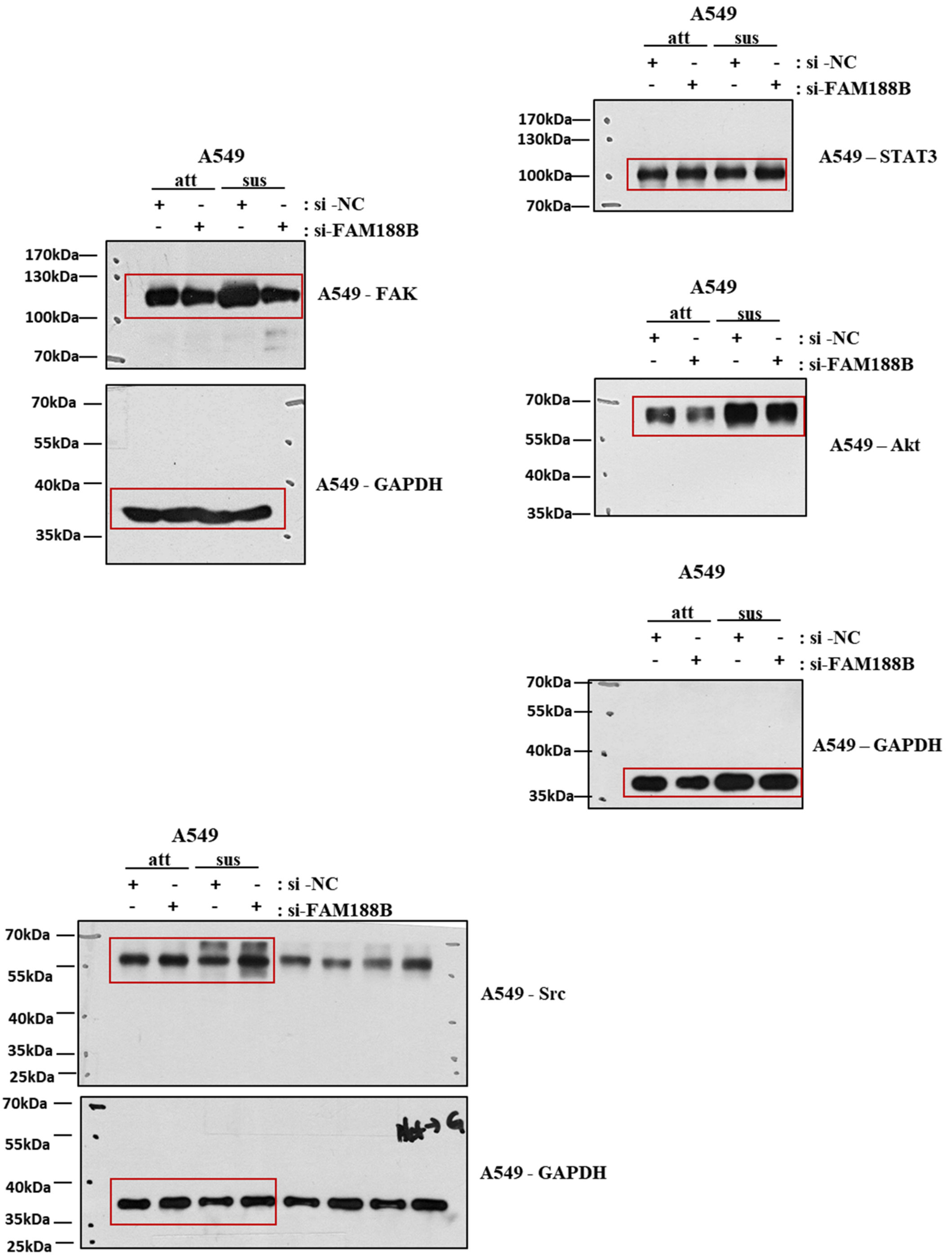

Supplementary figure 6B

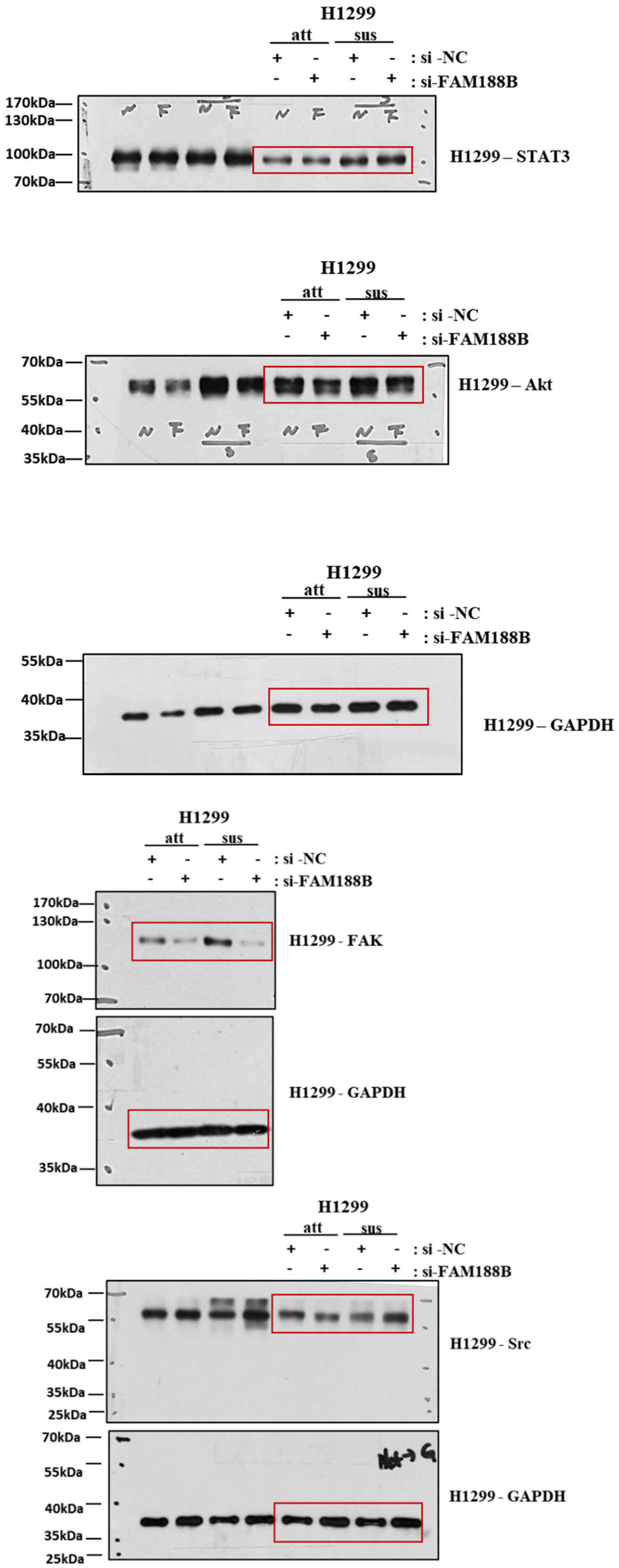

Supplementary figure 6C

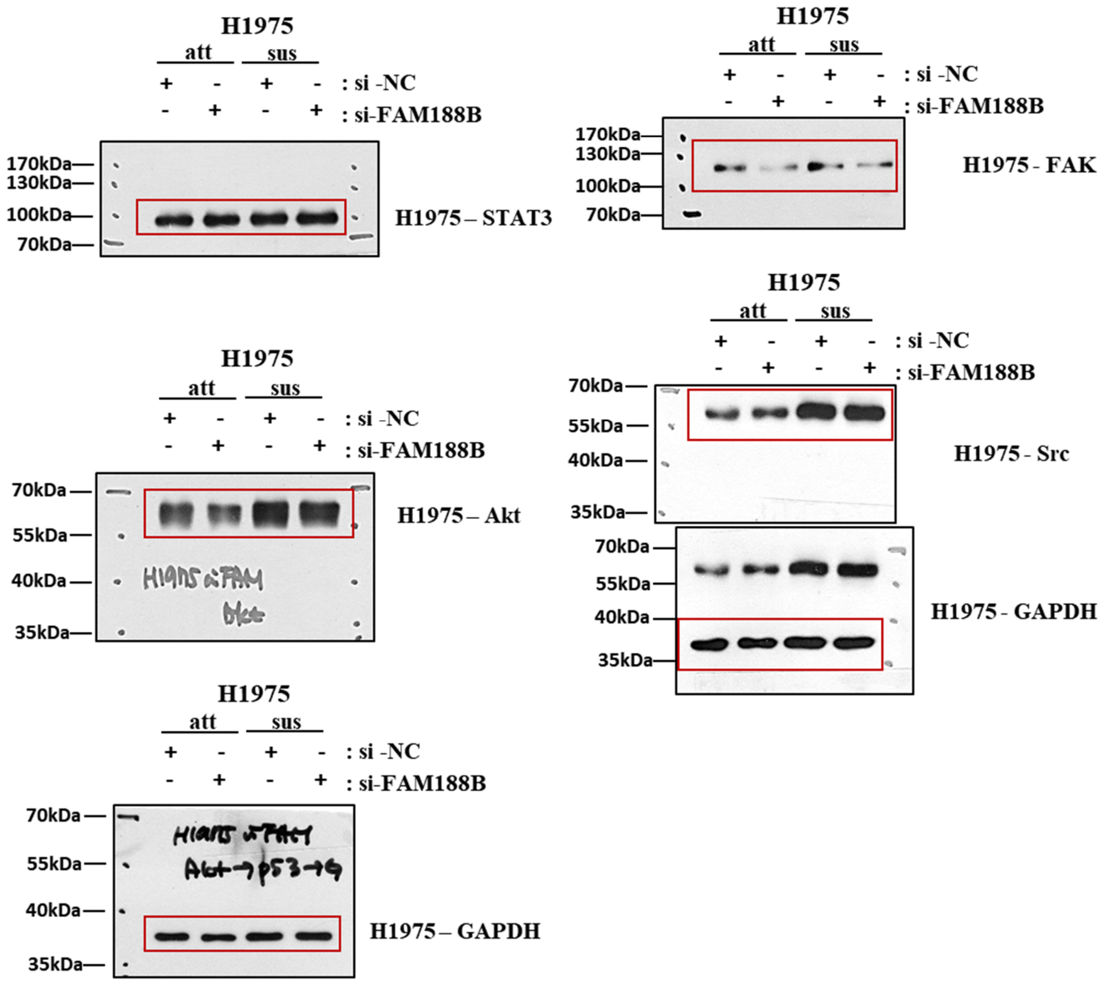

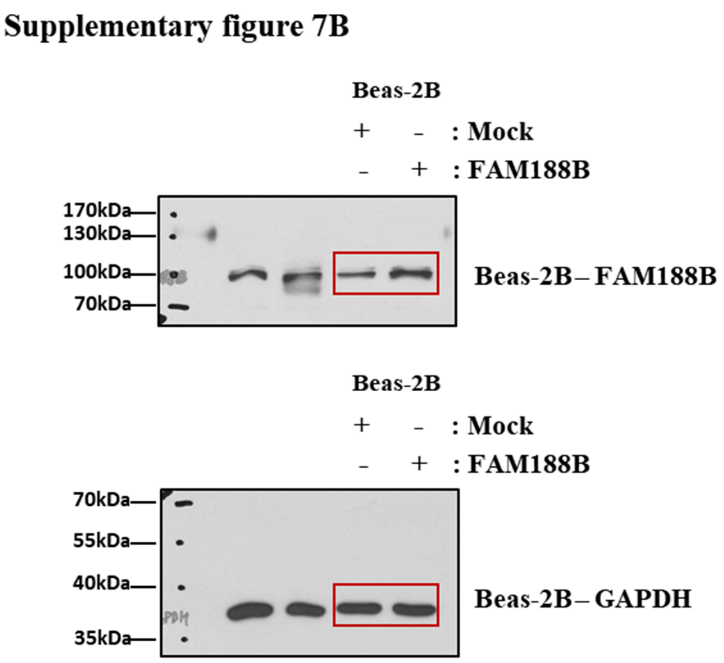

Figure S8. Original Western blot.
